# Hybrid Convolutional Vision Transformer for Robust Low-Channel sEMG Hand Gesture Recognition: A Comparative Study with CNNs

**DOI:** 10.3390/biomimetics10120806

**Published:** 2025-12-03

**Authors:** Ruthber Rodriguez Serrezuela, Roberto Sagaro Zamora, Daily Milanes Hermosilla, Andres Eduardo Rivera Gomez, Enrique Marañon Reyes

**Affiliations:** 1 Department of Mechatronics Engineering, University Corporation of Huila, Neiva 410001, Colombia; 2 Department of Mechanical Engineering, Universidad de Oriente, Santiago de Cuba 90500, Cuba; 3 Department of Automatic Engineering, University of Oriente, Santiago de Cuba 90500, Cuba; 4 Master’s Program in Artificial Intelligence, School of Engineering and Technology, Universidad Internacional de La Rioja (UNIR), 26006 Logroño, Spain; 5 Center for Neuroscience, Image and Signal Processing (CENPIS), Universidad de Oriente, Santiago de Cuba 90500, Cuba

**Keywords:** convolutional neural network (CNN), hand gesture recognition, hybrid deep learning, myoelectric pattern recognition, surface electromyography (sEMG), Vision Transformer (ViT)

## Abstract

Hand gesture classification using surface electromyography (sEMG) is fundamental for prosthetic control and human–machine interaction. However, most existing studies focus on high-density recordings or large gesture sets, leaving limited evidence on performance in low-channel, reduced-gesture configurations. This study addresses this gap by comparing a classical convolutional neural network (CNN), inspired by Atzori’s design, with a Convolutional Vision Transformer (CViT) tailored for compact sEMG systems. Two datasets were evaluated: a proprietary Myo-based collection (10 subjects, 8 channels, six gestures) and a subset of NinaPro DB3 (11 transradial amputees, 12 channels, same gestures). Both models were trained using standardized preprocessing, segmentation, and balanced windowing procedures. Results show that the CNN performs robustly on homogeneous signals (Myo: 94.2% accuracy) but exhibits increased variability in amputee recordings (NinaPro: 92.0%). In contrast, the CViT consistently matches or surpasses the CNN, reaching 96.6% accuracy on Myo and 94.2% on NinaPro. Statistical analyses confirm significant differences in the Myo dataset. The objective of this work is to determine whether hybrid CNN–ViT architectures provide superior robustness and generalization under low-channel sEMG conditions. Rather than proposing a new architecture, this study delivers the first systematic benchmark of CNN and CViT models across amputee and non-amputee subjects using short windows, heterogeneous signals, and identical protocols, highlighting their suitability for compact prosthetic–control systems.

## 1. Introduction

The classification of hand gestures using surface electromyography (sEMG) has become a key field in prosthetic control, human–machine interfaces, and rehabilitation technologies. This type of signal enables the non-invasive recording of electrical activity from superficial muscles, providing a direct pathway for decoding the user’s motor intentions [1]. With the advancement of deep learning techniques—particularly convolutional neural networks (CNNs)—significant improvements have been achieved in the automatic extraction of features and in the robust classification of high-dimensional sEMG data [2,3].

Beyond these applications, the versatility of sEMG has driven its integration into increasingly diverse fields. It is widely employed in brain–computer interfaces (BCIs) for the restoration of motor functions, in physical rehabilitation systems such as assisted exoskeletons for movement training, in contexts of collaborative robotics and interactive gaming, as well as in the control of wearable devices such as haptic gloves and lightweight exoskeletons. A recent study highlights the potential of sEMG for decoding movement intentions and enhancing human–robot interaction in upper-limb exoskeleton-assisted rehabilitation systems [4].

In the broader domain of biomedical signal processing, recent advances in artificial intelligence have markedly expanded the scope of deep-learning models beyond imaging and language tasks to complex physiological signals. For example, Lee et al. provides a comprehensive review of AI methods for biomedical signals, highlighting the integration of supervised, unsupervised and reinforcement-learning strategies in analyzing electromyograms, electroencephalograms and cardiovascular recordings [5]. Jeong et al. further explore machine-learning pipelines for wearable biosensors and demonstrate that model choice must account for signal characteristics, latency constraints and device-level deployment requirements [6]. Anwar et al. focus specifically on transformer architectures in biosignal analysis, offering a roadmap where self-attention mechanisms are applied to biosignals such as EMG and ECG and comparing them with recurrent and convolutional baselines [7]. Gopi presents a structured survey of lightweight transformers for ECG, EEG and EMG, examining model size, FLOPs, latency and the trade-offs necessary for on-device inference [8]. Moreover, Farmani et al. propose a multimodal transformer framework for neuromuscular gesture classification, showing that fusing multiple sensor modalities (e.g., HD-sEMG and accelerometry) can yield substantial performance gains in gesture recognition tasks [9]. Collectively, these findings support the view that architectures capable of modeling long-range temporal dependencies, cross-channel interactions and resource-efficient inference are increasingly critical in the design of next-generation assistive and prosthetic systems, thus providing a compelling rationale for applying hybrid convolutional–transformer designs to low-channel sEMG environments, as investigated in this work.

Several families of deep learning models have become established in the field of gesture recognition based on sEMG. Early convolutional architectures paved the way by exploiting local spatio-temporal correlations directly from the signal or from time–frequency representations. The seminal work by Atzori et al. demonstrated that well-designed convolutional architectures could compete with traditional feature-engineering schemes on datasets such as NinaPro, establishing a widely replicated benchmark within the community [10]. Along this line, efforts to compress CNNs for resource-constrained contexts have shown that low-complexity deep networks (e.g., EMGNet) can achieve high accuracies both on datasets acquired with the Myo armband and on the NinaPro benchmark, while reducing the number of parameters and training cost—an essential aspect for embedded applications [11].

In studies involving able-bodied subjects (NinaPro DB1/DB2, BioPatRec, CapgMyo, CSL-HD), hybrid CNN–RNN or CNN–LSTM architectures have reported consistent improvements (≈87–94%) by combining spatial feature extraction and temporal modeling with attention mechanisms [12]. However, in amputee populations, the evidence remains more limited and is mainly concentrated on datasets such as NinaPro DB3, where inter-subject variability and model generalization pose additional unresolved challenges. More recently, lightweight variants optimized for real-time operation have confirmed that spatio-temporal fusion enhances robustness in short analysis windows [13]. Likewise, transfer learning approaches applied to CNNs have increased accuracy in scenarios with limited data, while domain adaptation techniques have improved inter-subject generalization—one of the major challenges in sEMG-based recognition [14].

In parallel, Transformers have emerged as a competitive alternative for sEMG-based gesture recognition. Originally proposed in the field of natural language processing, these models rely on self-attention blocks that capture long-range dependencies within sequences without requiring recurrence. One of their most relevant adaptations to the domain of muscular signals is the Vision Transformer (ViT), which applies attention mechanisms to segmented signal representations. The CT-HGR model demonstrated that a ViT trained from scratch can classify up to 65 gestures in HD-sEMG recordings with 32–128 channels and time windows of 31–250 ms, outperforming 3D-CNN and SVM/LDA models in both accuracy and efficiency [15]. Moreover, variants integrating spike-train information (BSS) have shown that combining macroscopic and microscopic signals enhances classification performance. More recently, temporal Transformers employing depthwise convolutions have been proposed to strengthen long-range dependency modeling without compromising computational efficiency, extending their applicability beyond HD configurations [14].

Finally, hybrid CNN–ViT architectures (CViT) have gained increasing attention, in which a CNN serves as an initial convolutional block to extract local features and reduce dimensionality before the multi-head self-attention mechanism. In the context of sEMG, Shen et al. [16] demonstrated that this design outperforms pure CNNs by combining local robustness with global modeling. More recent studies on multichannel biosignals have further confirmed that CNN–Transformer fusion enhances both accuracy and generalization in inter-subject scenarios with moderately sized datasets.

Despite these advances, CNN-based methods still present limitations arising from their fixed receptive field and their reduced ability to capture long-range dependencies in temporal sequences, which restricts their adaptability to dynamic and heterogeneous signals [17,18]. In response, more complex hybrid architectures have been proposed, such as U-Net models with MobileNetV2 and BiLSTM encoders optimized through metaheuristic algorithms, which have demonstrated improvements in edge-computing environments [1], or multiscale architectures employing transfer learning to enhance generalization capability on the NinaPro dataset [19].

In parallel, Transformer-based models—and particularly Vision Transformers (ViTs)—have sparked growing interest in the biomedical domain. Originally designed for image processing tasks, ViTs divide the input into patches and apply attention mechanisms that enable the modeling of global relationships [20]. In the sEMG domain, several recent studies have explored this approach: TEMGNet, a ViT trained on NinaPro DB2, achieved over 82% accuracy with a reduced number of parameters [21]; EMGTFNet, which incorporates fuzzy logic blocks within a ViT framework, reached an average accuracy of 83.6% for the classification of 49 hand gestures in able-bodied subjects without requiring artificial data augmentation [22]; and BSS-CViT, which combines Transformer architectures with blind source separation techniques, achieved 96.6% accuracy on HD-sEMG recordings [23]. Complementarily, hybrid architectures such as TraHGR—based on multiple parallel Transformers—have demonstrated substantial improvements in robustness and performance on the NinaPro DB2 dataset [24].

These advances highlight an ongoing debate: while CNNs remain efficient and practical for small-scale datasets, ViTs and their variants offer greater capacity to model spatio-temporal dependencies and to generalize when trained on enriched signal representations, such as time–frequency spectrograms or spatial maps derived from HD-sEMG. Nevertheless, most existing studies focus on high-density configurations (HD-sEMG) or on large gesture sets, leaving a gap in knowledge regarding the comparative performance of CNNs and ViTs in scenarios with limited sensors and a reduced number of gestures.

This work addresses this gap by conducting the first systematic benchmark of CNN and Vision Transformer (ViT) models under low-channel sEMG conditions in amputee and non-amputee populations. Rather than introducing a novel architectural block, the proposed approach adapts a hybrid CNN–ViT design in which a convolutional stem derived from Atzori et al. is used as a domain-specific local feature extractor prior to Transformer encoding. This adaptation facilitates robust tokenization and attention modeling on short sEMG windows without requiring spectrograms or high-density spatial mappings. Two datasets were evaluated: a proprietary Myo-based collection (10 subjects, 8 channels, six gestures) and a subset of NinaPro DB3 (11 transradial amputees, 12 channels, same gestures). All experiments were conducted following a subject-specific protocol with multiple repetitions per participant, enabling controlled and reproducible comparisons. The results demonstrate that despite the limited number of channels, the hybrid CNN–ViT approach consistently matches or outperforms the classical CNN baseline, highlighting its potential for compact, low-sensor prosthetic control systems.

Therefore, the central aim of this study is to conduct a systematic comparison between a classical CNN architecture and a hybrid CNN–Vision Transformer (CViT) under low-channel sEMG conditions in both amputee and non-amputee populations. Our working hypothesis is that the CViT model will exhibit superior robustness and generalization, particularly in amputee recordings characterized by low signal-to-noise ratio, high inter-subject variability, and limited spatial redundancy. To test this hypothesis, we evaluate both models across two complementary datasets using standardized preprocessing, balanced segmentation, and subject-specific experimental protocols.

## 2. Materials and Methods

### 2.1. Datasets

#### 2.1.1. NinaPro DB3 Database

The NinaPro DB3 dataset, recorded using a Delsys Trigno sensor system (Delsys Inc., Natick, MA, USA) at a sampling frequency of 2 kHz, contains electromyographic (EMG) data from 11 transradial amputee subjects. These participants performed 52 hand-grasp postures, with EMG signals captured through 12 sensors strategically placed on the forearm to measure the muscular activity associated with each movement [25]. The demographic and clinical characteristics of the participants are summarized in Table 1 [26].

**Figure 1:**
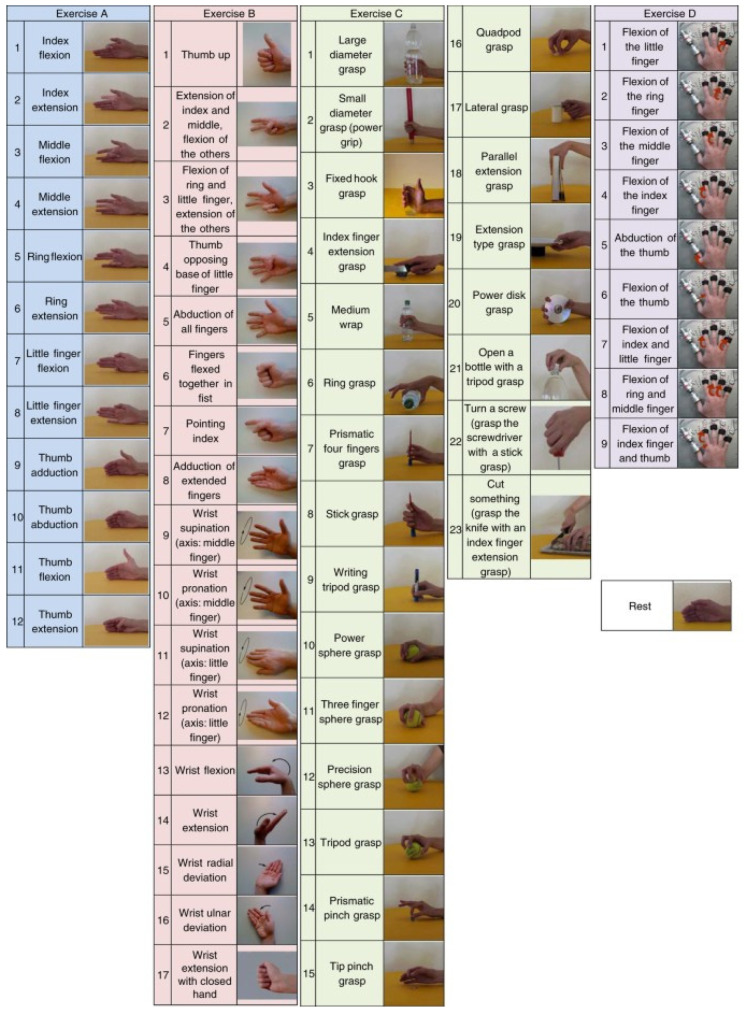
Exercise-Wise Movements and Force Patterns. Reproduced from Refs. [25,26].

In this study, six specific postures were selected from the original 52 (See Figure 1): power grasp, palm inward, palm outward, open hand, pinch grasp, and rest. These gestures were chosen because they represent functional grasps commonly used in daily prosthetic control and correspond directly to the gesture vocabulary recorded by the Myo armband. Selecting an identical gesture set across both datasets ensures comparability and reflects realistic prosthetic control scenarios, as supported by prior studies that restrict NinaPro DB2/DB3 to functional grasp subsets for low-channel evaluation (e.g., Atzori et al., [25]). Although this reduction simplifies gesture space, it avoids introducing artificial inconsistencies between datasets and maintains ecological validity.

This focus on six postures facilitates the analysis and classification of EMG signals, allowing the classification models to concentrate on a manageable set of movements while maintaining the quality of the data captured in the NinaPro DB3 dataset. This provides a solid foundation for developing accurate and efficient algorithms for motion recognition in myoelectric prosthesis applications.

#### 2.1.2. Myo Armband Dataset

Twenty able-bodied subjects and ten amputee participants, both male and female, aged between 24 and 65 years, took part in the experiments. All procedures were conducted in accordance with the Declaration of Helsinki and were approved by the Ethics Committee of the University of Tolima (approval number: N–20160021). All participants volunteered and provided written informed consent prior to the experimental procedures. In the experiments reported in this study, only the ten amputee participants recorded using the Myo device (Myo Armband, Thalmic Labs Inc., Waterloo, ON, Canada) (the “Myo–Amputee” cohort) were included, while the recordings from able-bodied subjects were excluded from the analysis.

All amputee participants with prior experience in the use of hand prostheses will be included in the study, with their background in using either passive or myoelectric prosthetic devices recorded in advance.

Demographic and clinical aspects to be recorded include, for each subject, age, sex, height, weight, and level of education. In the case of amputee participants, additional information such as dominant hand, side of amputation, year and cause of amputation, type of prosthesis currently used or previously used, and level of amputation will also be documented.

- Inclusion Criteria:

Adults aged between 20 and 65 years were included in the study, with no history of neurological and/or psychiatric disorders, voluntary participation, and approval from the medical personnel involved in the experiments. For amputee participants, only those with transradial amputation levels were considered; individuals with amputations above the elbow or beyond the wrist were not admitted to the study. Any deviation from these parameters constituted exclusion criteria. Table 2 presents the characteristics of the amputee patients who participated in the experiments. The DASH index is a self-administered 30-item questionnaire designed to assess the patient’s health status during the preceding week. It evaluates the upper limb as a functional unit, allowing the quantification and comparison of the impact of different conditions affecting various regions of the limb, the higher the score, the greater the disability.

It is important to acknowledge that the Myo–Amputee dataset includes only ten transradial amputee participants. Although this sample size is consistent with several previous clinical sEMG studies involving amputees, it remains limited for deep learning applications. As a result, the evaluation conducted here reflects within-subject characteristics rather than broad population-level generalization. In the Discussion, we further address the implications of this limitation and its impact on model robustness.

#### 2.1.3. Myo Armband sEMG Sensor

The data were recorded using the commercial MYO armband (Thalmic Labs Inc., Waterloo, ON, Canada) (MYB). The MYB is a portable sEMG sensor developed by Thalmic Labs, featuring eight dry-electrode channels with a sampling frequency of 200 Hz. It is a low-cost, consumer-grade device equipped with a nine-axis inertial measurement unit (IMU) [27] that communicates wirelessly with a computer via Bluetooth. The device is non-invasive and easier to use compared to conventional electrodes [28,29]. Despite its relatively low sampling frequency, previous studies have demonstrated that its performance is comparable to full-band EMG recordings obtained with conventional electrodes [11,30], and the technology has been employed in numerous research applications [31]. Therefore, this study did not focus on comparisons with conventional sEMG electrodes.

The software developed [32] was primarily designed for the acquisition and real-time visualization of electromyographic (sEMG) signals, enabling the analysis of muscular activity in various contexts such as medical research, biomechanics, and device control. From its conception, the focus was on creating an intuitive and efficient tool capable of accurately recording data and exporting it in multiple formats for more detailed analysis.

**Figure 2:**
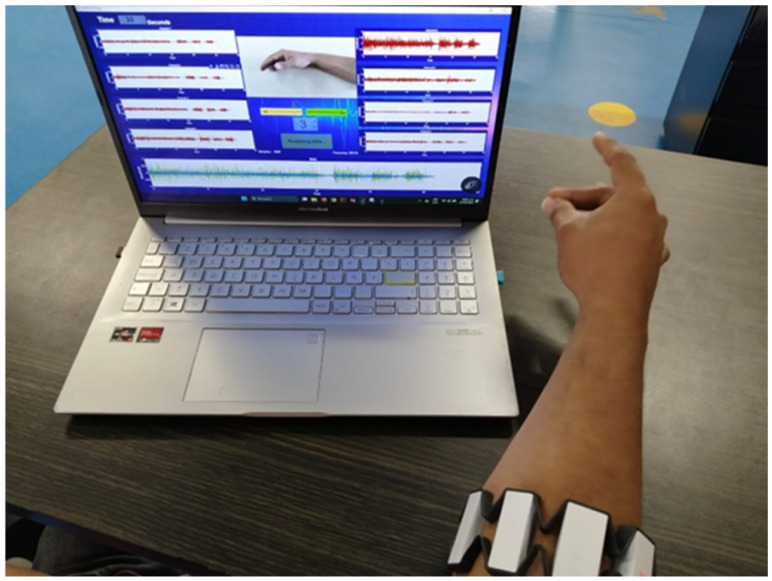
sEMG Signal Acquisition with the MATLAB-Based Application. Reproduced from Ref. [32].

During its development, the software was used to monitor muscular patterns in real time, visualize multiple sEMG channels simultaneously, and classify signals with high accuracy. Additional features were implemented to capture images of the generated plots and facilitate subsequent analysis. Furthermore, compatibility was integrated with devices such as the Myo Armband sensor, following an experimental protocol that ensures consistency in data acquisition (see Figure 2). As a result, it was possible to record sessions involving specific hand postures, obtaining key information to evaluate muscular activity across different scenarios.

**Figure 3:**
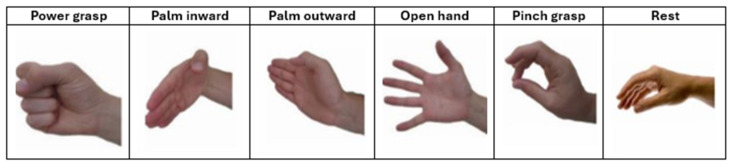
Gestures Recognized by the MYO Device.

The armband was configured to identify six basic movements essential for improving grasp performance: power grasp (AP), palm inward (PI), palm outward (PO), open hand (MO), pinch grasp (AT), and rest (RE) (see Figure 3).

**Figure 4:**
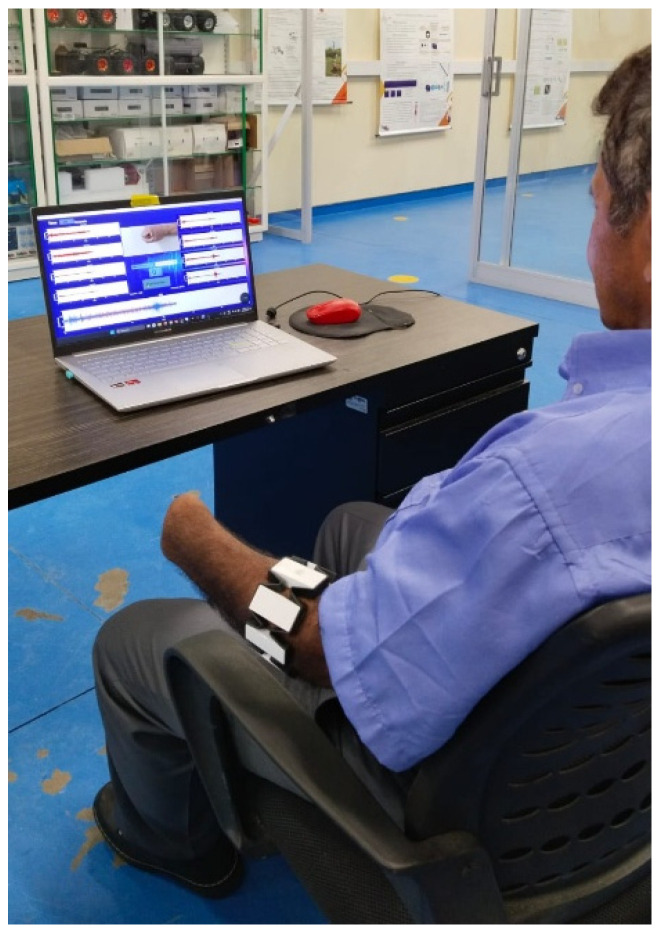
Transradial Amputation Patient.

- sEMG Signal Recording:

Prior to the experimental sessions, the subjects were instructed on the experimental procedure, and as a first step, the armband was calibrated for both limbs. During the recording of each gesture, participants sat comfortably in front of the computer with both elbows flexed at 90 degrees and were instructed to perform the gestures displayed on the monitor—using the contralateral limb and the amputated limb in the case of amputee participants (see Figure 3 and Figure 4).

The graphical interface provided the participants with timing cues for performing the tests and for rest periods (see Figure 4). Recordings for amputee participants were conducted in repeated sessions over the course of one week.

**Figure 5:**
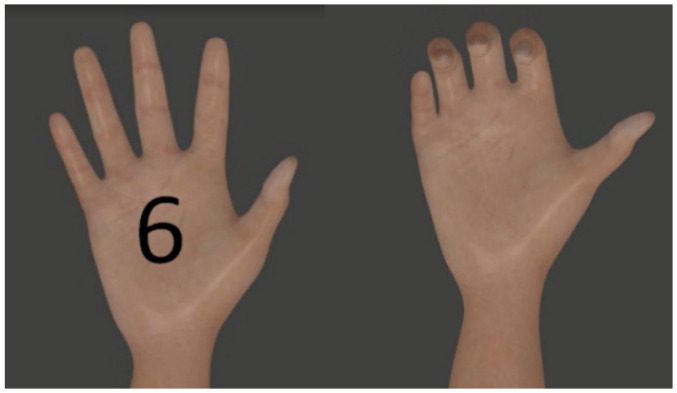
User interface displaying the imaginary hand movement to be performed, along with execution and rest periods.

The experimental procedure for capturing the myoelectric signals was as follows: for each hand gesture or grasp, 200 samples were collected over a 30 s interval. Transitions between each of the six proposed gestures were performed with 1 min rest intervals, as recommended in [10] (See Figure 5). Recordings were carried out across multiple sessions and on different days of the week. The data from these myoelectric signals were stored in matrices for subsequent offline processing.

### 2.2. sEMG Signal Preprocessing

The preprocessing of surface electromyographic (sEMG) signals is a crucial stage to ensure the quality and consistency of the data before their use in deep learning models. In this study, specific preprocessing procedures were developed for each dataset used (Myo and NinaPro), while maintaining a comparable methodological workflow.

#### 2.2.1. Custom sEMG Dataset Collected for This Study

The proprietary dataset was acquired using the Myo Armband, which consists of eight dry-electrode channels with a sampling frequency of 200 Hz. Once the signals were captured, they were exported in CSV format for subsequent offline processing.

Initially, the eight channels were standardized by class, applying a scaling procedure that adjusted the values to zero mean and unit variance. This process aimed to compensate for variability introduced by factors such as device placement, muscle contraction intensity, and physiological differences among subjects, thereby reducing dispersion across gestures and facilitating the classification task.

(1) $$\begin{array}{c} x_{i,j}^{\prime }=\frac{x_{i,j}-\mu_{c,j}}{\sigma_{c,j}}\; \end{array}$$

where $x_{i,j}$ corresponds to the original value of channel j in sample $i,\;\mu_{c,j}$ represents the mean of channel j for class $c,\;\sigma_{c,j}$ is the standard deviation computed for the same class and channel, and $x_{i,j}^{\prime }$ is the resulting value after standardization.

Subsequently, the signals were segmented into fixed-size temporal sliding windows with a certain degree of overlap between them. This step aimed to preserve the temporal dynamics of muscle contractions while simultaneously increasing the number of samples available for training. Only windows containing samples from a single gesture were retained, ensuring class purity and preventing the introduction of noise from transitions between movements.

(2) $$\begin{array}{c} X_{k}=\left\{x_{k}.\left(W-O\right)+1,x_{k}.\left(W-O\right)+2,\ldots ,x_{k}.\left(W-O\right)+W\right\} \end{array}$$

where $X_{k}$ corresponds to the k, W is the window size expressed in number of samples, O represents the number of overlapping samples between consecutive windows, and $x_{t}$ denotes the sEMG signal value at time t. This procedure allows capturing the temporal evolution of the signal, ensuring continuity of information and reducing data loss at the boundaries of each segment.

Once segmented, the windows were randomly shuffled together with their corresponding labels to break the temporal dependency of the sequences and ensure that the models did not memorize local patterns associated with the acquisition order.

Finally, the preprocessed windows were transformed according to the deep learning architecture to be used:

- In the case of the CNN, the windows were reorganized into two-dimensional matrices that preserve the relationship between channels and samples, allowing the use of spatial convolution operations to detect discriminative patterns.
- In the case of the ViT, each window was treated as a sequence of vectors and subsequently embedded into fixed-dimensional representations. These representations were divided into temporal “patches,” which serve as the input to the multi-head self-attention mechanism block responsible for modeling long-range dependencies among signals from the different channels.

This unified preprocessing workflow ensures that, despite architectural differences, the models operate on comparable and consistent representations, facilitating a fair evaluation of their classification capabilities under homogeneous conditions.

#### 2.2.2. NinaPro DB3 Dataset

For this study, data from the NinaPro dataset in MATLAB (.mat) format were used. Since this dataset includes multiple classes and repetitions of movements, a custom Python script was developed to select the classes of interest for the analysis. The chosen gestures were power grasp (AP), palm inward (PI), palm outward (PO), open hand (MO), pinch grasp (AT), and rest (RE).

**Figure 6:**
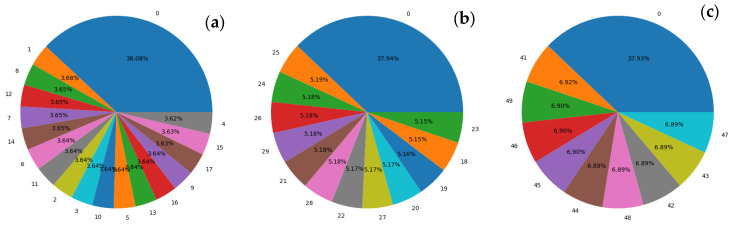
Distribution of the NinaPro Dataset. (**a**) exercise 1, (**b**) exercise 2, (**c**) exercise 3.

In the NinaPro dataset, these gestures correspond to identifiers 0, 6, 13, 14, 5, and 2, respectively (see Figure 6). Additionally, the stimulus repetition index column was included, indicating the repetition of each movement within a recording session. This information is essential to ensure that the captured signals are representative and can be reliably used for model validation and testing.

To ensure a fair evaluation, the data were balanced so that all classes contained the same number of samples. This process helps prevent bias during model training, improving both accuracy and generalization capability. Finally, the processed and balanced data were converted from MATLAB (.mat) format to CSV (Comma-Separated Values) format, thereby facilitating data handling throughout the study.

##### Low-Pass Filter

To attenuate high-frequency noise and preserve physiologically relevant features of the sEMG signal, a multi-stage low-pass Butterworth filter was implemented. Following previous approaches reported in the literature [33], this filter was applied in three iterations with orders of 1, 3, and 5, allowing the capture of different levels of signal smoothing. As a result of this process, a three-channel signal was generated, where each channel represents the output of the filtered signal at a specific filter order. The combination of these channels enhances the representativeness of the preprocessed signal, facilitating the extraction of discriminative features in the classification stage.

##### Normalization

Since sEMG signals exhibit considerable variability due to differences in electrode placement, muscle contraction intensity, and physiological variations among subjects, a normalization technique is required to ensure signal stability. For this purpose, µ-law normalization is employed—an approach originally used for audio signal compression and later adapted for sEMG signals. This normalization is defined by the following equation:

(3) $$\begin{array}{c} F\left(x_{t}\right)=\mathrm{sign}\left(x_{t}\right)\frac{\ln\left(1+\mu \left|x_{t}\right|\right)}{ln\left(1+\mu \right)} \end{array}$$

where ***x*** represents the signal amplitude at time ***t*** and ***μ*** is a tuning parameter that controls the scale of the transformation. This procedure mitigates both inter-subject and intra-subject variations, thereby improving the generalization capability of the machine learning model.

##### Segmentation

To ensure proper model evaluation and prevent overfitting, the dataset was divided into two subsets: training and testing. This process was performed using the movement repetition index stored in the Repetition column of the dataset.

During sEMG signal acquisition, each gesture was repeated multiple times by the subjects, introducing variability in the captured signals. To ensure a more realistic evaluation and allow the model to handle previously unseen data, specific repetitions were selected for each subset (See Table 3):

- Training Set:

Repetitions 1, 3, 4, and 6 were used. This subset contains the samples that the model uses to learn the relationships between the signal features and the corresponding gesture labels.

- Testing Set:

Repetitions 2 and 5 were used, reserved exclusively for model evaluation. These data were not used during training, allowing the measurement of the model’s actual performance when faced with gestures performed under conditions different from those seen during training.

The segmentation of the signals in the NinaPro dataset was performed using the same procedure previously described for the Myo dataset (Equation (2)). In this case, the signals were divided into sliding windows of 50 samples with an overlap of 15 samples, allowing continuous capture of the temporal evolution of each gesture. The selection of the window sizes and overlap ratios followed dataset-specific constraints related to sampling frequency and physiological characteristics. For the Myo armband (200 Hz), a 30-sample window (~150 ms) is consistent with recommended durations for low-density sEMG intended for real-time prosthetic control, capturing a complete motor unit action potential firing cycle while avoiding excessive temporal smoothing. For NinaPro DB3 (2 kHz), a 50-sample window (~25 ms) preserves fine-grained temporal variations required in higher-resolution recordings, enabling accurate modeling of rapid muscle activation changes. The overlap settings were chosen to balance temporal continuity and computational cost: 50% in Myo to compensate for its lower sampling rate, and ~30% in DB3 to limit redundancy while maintaining class separation. These values align with common segmentation practices reported in sEMG literature and ensure that both CNN and CViT models operate on physiologically meaningful and comparable temporal units. As in the proprietary dataset, only windows associated with a single gesture were retained, thereby ensuring class purity and avoiding the inclusion of transitions between movements. This segmentation scheme guarantees homogeneity in the preprocessing of both datasets, facilitating a direct comparison of model performance.

After completing the segmentation and standardization process, the resulting windows were adapted to the corresponding deep learning architecture. In the case of the CNN, each window was organized into a two-dimensional matrix, allowing the convolutions to capture spatio-temporal patterns across channels and consecutive samples. For the ViT, the same windows were transformed into sequences of embedded vectors, which were subsequently divided into temporal patches and processed through multi-head self-attention mechanisms. This strategy ensures that, despite architectural differences, both models operate on comparable representations of the sEMG signals, enabling a fair and homogeneous evaluation of their performance.

It is important to clarify that the evaluation protocol used in this study is subject-specific. All training and testing samples belong to the same participant, and only different repetitions were kept independent. No cross-subject splitting was performed, and therefore the results do not assess generalization to unseen subjects.

### 2.3. Proposed Models

The windows generated during preprocessing serve as the common input for the two deep learning architectures evaluated in this study: a convolutional neural network (CNN), based on the approach of Atzori et al. [10], and a Vision Transformer (ViT) implemented in TensorFlow. Both architectures were selected with the aim of comparing a classical model, widely used in myoelectric gesture recognition, with a more recent approach that leverages self-attention mechanisms.

#### 2.3.1. Convolutional Neural Network (CNN)

**Figure 7:**
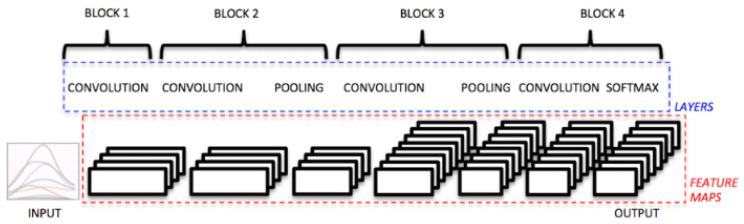
Structure of the Convolutional Network Proposed by Atzori. Reproduced from Ref. [10].

The convolutional architecture implemented in this work was designed following the approach proposed by Atzori et al. [10] (see Figure 7), adapted to the dimensions and characteristics of the datasets used (Myo and NinaPro DB3). In both cases, the segmented sEMG signal windows were structured as two-dimensional tensors (channels × time), allowing simultaneous exploitation of the spatial correlations between channels and the temporal dynamics of muscle contractions.

The specific configuration of each model is summarized in Table 4. As observed, the architecture maintains the same structure across both datasets: four convolutional layers interleaved with ReLU activations and average pooling operations, followed by a flattening layer and a dense layer with a softmax activation, responsible for assigning probabilities to the six gesture classes. The main difference lies in the input dimensions: in the Myo dataset, the windows have a size of 8 × 30 × 1, whereas in NinaPro DB3 they reach 8 × 50 × 1 due to differences in the temporal segmentation applied during preprocessing.

Each convolutional layer was designed to capture different types of patterns. The initial kernels (1 × 12 and 3 × 3) focus on local dependencies and on the extraction of elementary signal features, whereas the larger kernels (5 × 5 and 9 × 1) enable the network to capture longer-range relationships by combining extended temporal information with spatial interactions across channels. This hierarchical design allows the network to progressively extract higher-level and more discriminative descriptors.

The CNN training was performed using the Stochastic Gradient Descent (SGD) optimizer with an initial learning rate of 0.001 and a momentum of 0.9. The loss function employed was categorical cross-entropy, which is appropriate for multiclass classification problems. Training was limited to a maximum of 100 epochs with a batch size of 15. To prevent overfitting, several regularization strategies were implemented, including early stopping (with a patience of 15 epochs), ReduceLROnPlateau (adaptive reduction in the learning rate when the validation error stagnates), and ModelCheckpoint (saving the best-performing model in each run).

Additionally, the experimental scheme incorporated stratified cross-validation (k = 5), ensuring that the class distribution was preserved within each fold. This procedure enabled the generation of more robust performance estimates and reduced the variance associated with specific data splits. For the Myo dataset, 14 experimental repetitions were conducted per subject, while the same procedure was applied to the NinaPro DB3 dataset, thereby maintaining the comparability of results.

#### 2.3.2. Convolutional Vision Transformer (CViT)

The proposed CViT is not a novel architecture in itself; rather, it represents a domain-adapted implementation of a hybrid CNN–ViT pipeline tailored for low-channel sEMG. In our design, a lightweight convolutional stem—derived from the architecture of Atzori et al.—is used as a local temporal feature extractor prior to Transformer encoding. This adaptation is motivated by the challenges inherent to amputee and low-density sEMG recordings, including low signal-to-noise ratio (SNR), high inter-subject variability, and minimal spatial redundancy across channels. By reducing dimensionality and emphasizing robust short-window representations, the convolutional stem facilitates stable patch embedding and effective attention modeling without relying on spectrograms or high-density spatial maps. A relevant example is the ViT-HGR (Vision Transformer for Hand Gesture Recognition), which employs an architecture designed from scratch for HD-sEMG signals (65 isometric gestures in healthy subjects, 32–128 channels). It demonstrated that pure Transformer models can achieve robust classification performance without the need for data augmentation or transfer learning, using a compact model (~78,210 parameters) that reached an average accuracy of 84.6% ± 3.1% with 31 ms windows [34]. Although structurally powerful, this approach entails a high computational cost and requires high-density signal configurations.

In other biomedical domains, hybrid CNN–ViT models have begun to be employed, where a convolutional network acts as a feature extractor preceding the Transformer block. This approach reduces dimensionality and focuses attention on more abstract representations. For example, in medical imaging studies, improvements of 5–7% have been reported in metrics such as accuracy and sensitivity when combining CNN and ViT architectures, compared to using standalone models [35].

Although CNN–Transformer hybrids have begun to show promising results in biomedical domains and signals from able-bodied subjects, no robust study has yet been reported that applies this architecture specifically to amputee populations for sEMG-based gesture recognition. Some related works (e.g., CNN + attention or CNN + RNN models) have been evaluated on datasets composed of healthy individuals or mixed groups with few amputees, but without a clear focus on this subgroup. This gap in the literature motivates and supports the need for our study on amputee populations using low-channel sensors.

#### 2.3.3. Proposed Architecture

The architecture implemented in this work corresponds to a Convolutional Vision Transformer (CViT) specifically designed for surface electromyography (sEMG) signals acquired in low-channel scenarios. Unlike most previous Transformer-based approaches developed for high-density configurations, our proposal is adapted to more portable settings, with only 8 channels in the Myo dataset and 12 in NinaPro DB3, processed in short temporal windows (30 and 50 samples, respectively). The model combines two main stages: an initial convolutional block (conv-stem), derived from the Atzori architecture, which acts as a local feature extractor and filters out redundancies inherent to biological signals; and a Transformer block, responsible for capturing global relationships and long-range dependencies within the embedded sequences. This integration leverages the efficiency of a lightweight conv-stem instead of a costly image-based pretraining, maintaining low computational overhead while ensuring robust representations that facilitate generalization under a subject-specific framework.

(1) Conv-stem (Local Feature Extraction)

The model receives as input the previously segmented and normalized sEMG signal windows:

- For the Myo dataset, the input is a tensor of size 8 × 30 × 1, corresponding to 8 muscle channels and 30 temporal samples per window.
- For the NinaPro DB3 dataset, the input has a size of 12 × 50 × 1, reflecting the 12 acquisition channels and the longer windows in the temporal domain.

In the implemented CViT, the convolutional architecture proposed by Atzori et al. [10]—previously described in the section above (Section 2.2.2)—is reused as the initial feature-extraction block. This decision is justified by the fact that the model has become a benchmark in myoelectric movement classification, balancing computational simplicity with high discriminative power in sEMG signals. When integrated as the conv-stem, the raw sEMG windows are transformed into a hierarchical activation map in which the first convolutional layers capture local temporal patterns and immediate inter-channel dependencies, while the deeper layers expand the receptive field and consolidate broader spatio-temporal relationships. This process provides the Transformer block with a more compact, stable, and abstract representation, reducing the redundancy and noise characteristic of biological signals. In this sense, the Atzori-based conv-stem functions as a specialized filter that refines and organizes the relevant information, ensuring that the subsequent Transformer encoder receives higher-quality inputs for modeling global dependencies.

(2) Embeddings and Attention (Transformer Encoder)

After the convolutional block, the output is reorganized into embedded patches that condense spatio-temporal fragments enriched by the previous convolutions. Each patch is projected through a linear layer into a fixed-dimension vector (d = 64), thus generating a homogeneous sequence of tokens. To ensure the model’s ability to capture global dependencies, two additional components are included:

- Class token: A learnable vector inserted at the beginning of the sequence that acts as a global descriptor of the entire window. During training, this token accumulates discriminative information from the remaining patches and ultimately feeds the classification head [15].
- Positional embeddings: Learnable vectors added to each token to preserve temporal order. Since Transformers lack an intrinsic notion of position, this encoding ensures that the temporal relationships inherent to the myoelectric signal are maintained [36].

**Figure 8:**
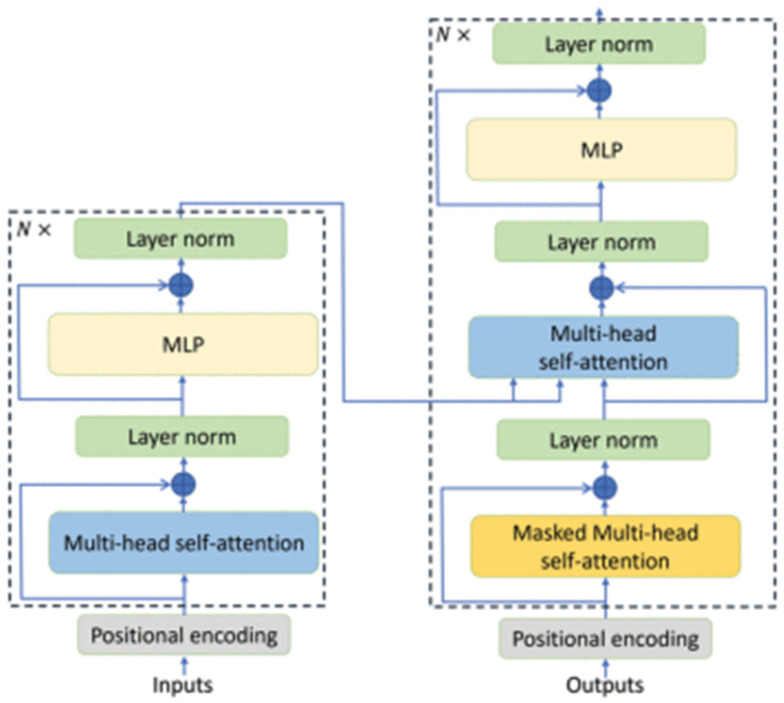
Transformer Encoder. Reproduced from Ref. [37].

Figure 8 illustrates the general architecture of the Transformer Encoder proposed by [37], which serves as the foundation for the block implemented in this work. As shown, each layer includes multi-head self-attention mechanisms, layer normalization, and residual feed-forward blocks—components that are also integral to the proposed CViT architecture.

The implemented Transformer encoder consists of four identical layers, each composed of the following modules:

1. Layer normalization (LayerNorm) applied to the input sequence.
2. Multi-head self-attention mechanism (MHSA) with eight heads (h = 8) and key dimension $d_{k}=64$. Each head evaluates global correlations among patches, identifying relevant regions at different scales. The scaled dot-product attention is defined as:

(4) $$\begin{array}{c} \mathrm{Attention}\left(Q,K,V\right)=\mathrm{softmax}\left(\frac{QK^{T}}{\sqrt{d_{k}}}\right)V\; \end{array}$$

where Q, K, and V represent the query, key, and value matrices, respectively [15,36].

3. Residual connection that preserves gradient stability and enhances information propagation.
4. Feed-Forward Block (MLP) consisting of two fully connected layers: the first with 256 units and GELU activation, followed by a dropout layer, and the second projecting back to the original dimension d = 64.
5. Second residual connection, which, together with normalization, stabilizes the learning process and accelerates convergence [38].

The multi-head self-attention process is formulated as:

(5) $$\begin{array}{c} \mathrm{MultiHead}\left(Q,K,V\right)=\mathrm{Concat}\left({head}_{1},\ldots ,{head}_{h}\right)W^{O}, \end{array}$$

with

(6) $$\begin{array}{c} {head}_{i}=\mathrm{Attention}\left(QW_{i}^{Q},KW_{i}^{K},VW_{i}^{V}\right), \end{array}$$

where $W_{i}^{Q},W_{i}^{k},W_{i}^{V}$ are learnable projection matrices for each head, and $W^{O}$ corresponds to the final output transformation [36].

The repetition of these layers in depth equips the model with the ability to integrate both short- and long-range dependencies. In this regard, the conv-stem provides robust local representations, while the Transformer encoder integrates these representations within a global attention framework. This combination has been demonstrated to enhance generalization in sEMG-based gesture recognition tasks, both in high-density configurations and in portable low-channel systems [15,36,38].

(3) Classification Block

The final stage of the architecture is designed to condense the global information processed by the Transformer encoder and transform it into a multiclass prediction. For this purpose, the class token—introduced at the beginning of the embedding sequence—is used. After passing through the attention layers, it accumulates the most relevant features from the remaining patches. In this way, the class token acts as a discriminative summary of the entire signal window, following an approach employed in recent ViT architectures applied to sEMG, such as CT-HGR [15].

The final representation of the class token is processed through an intermediate dense layer with GELU activation and dropout, which enhances the model’s nonlinearity and helps prevent overfitting. Subsequently, the output is directed to the softmax classification layer, consisting of six output nodes corresponding to the six evaluated hand gesture classes.

The output function is formalized as:

(7) $$\begin{array}{c} P\left(y=c|z\right)=\frac{\exp\left(z_{c}\right)}{\sum_{k=1}^{6}\exp\left(z_{c}\right)} \end{array}$$

where $z_{c}$ represents the activation associated with class ccc. This formulation ensures that the model outputs are interpreted as normalized probabilities, allowing each sEMG window to be assigned to a specific class within the gesture space.

The proposed CViT was trained under a carefully tuned configuration for sEMG signals in low-channel scenarios. Table 5 summarizes the main hyperparameters and practical decisions used during training, defined based on preliminary experimentation and recent references in the field. This level of detail ensures the reproducibility of the study and clarifies how training stability was balanced with computational efficiency. The encoder depth (four layers) was set as a compromise between modeling capacity and computational cost in short-window, low-channel conditions, thus avoiding overparameterization in low-dimensional signal contexts. Eight attention heads were adopted to increase the diversity of attended patterns without excessively expanding the parameter count, while an embedding dimension of 64 was chosen to maintain compact and numerically stable tokens in short sequences. The Adam optimizer with an initial learning rate of ${1\times 10}^{-3}$ was employed for its reliable convergence on non-stationary biosignals. A batch size of 32 balanced gradient stability and memory usage, and a maximum of 200 epochs allowed sufficient exploration of the solution space under a subject-specific scheme, with optimal model selection governed by the early stopping.

**Figure 9:**
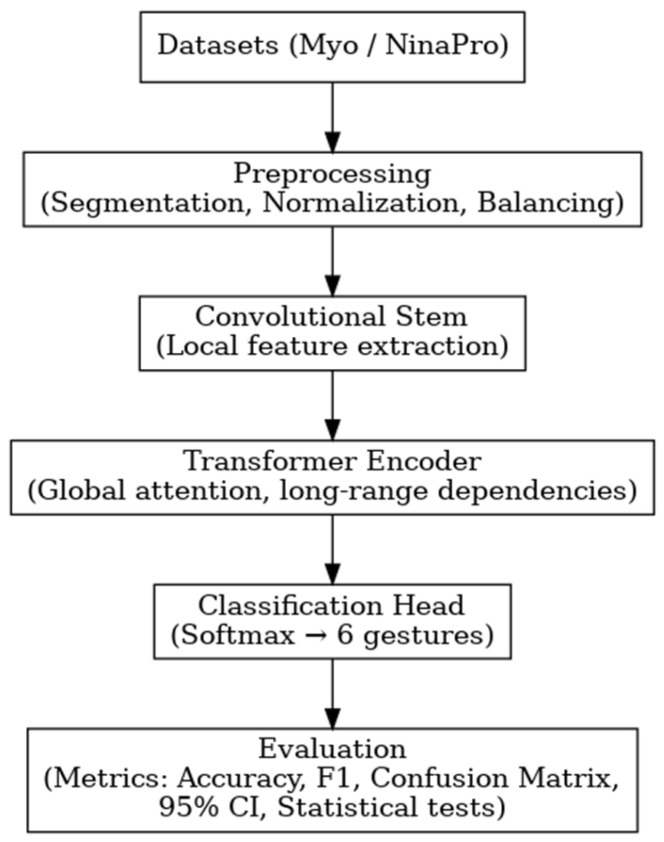
Methodological framework of the study: from sEMG signal acquisition and preprocessing to classification using the CViT model and performance evaluation.

The proposed CViT was integrated into a subject-specific framework, in which each participant contributed independent repetitions that were segmented into fixed windows (30 samples for Myo and 50 for NinaPro DB3) and subsequently normalized at the channel level. To prevent bias, the classes were balanced in each iteration through stratified sampling, ensuring an equitable representation of gestures. These normalized windows served as the direct input to the initial convolutional block, whose activations were transformed into embedded tokens for the Transformer encoder. Finally, the model outputs were evaluated using standard classification metrics and subjected to statistical tests, forming a complete data→CViT→metrics pipeline consistent with the subject-specific experimental design (see Figure 9).

### 2.4. Model Complexity and Computational Requirements

To assess the feasibility of deploying the proposed models in embedded and prosthetic applications, we quantified their computational complexity in terms of trainable parameters and estimated floating-point operations (FLOPs). Model summaries obtained from TensorFlow indicate that the classical CNN contains 99,398 parameters for the Myo dataset and 109,766 parameters for NinaPro DB3 (See Table 6).

In contrast, the hybrid CNN–ViT architecture contains 440,262 parameters (Myo) and 441,158 parameters (DB3), reflecting the additional cost introduced by multi-head self-attention.

FLOPs were estimated to use TensorFlow profiler tools. The CNN required approximately 8.1 MFLOPs (Myo) and ≈20 MFLOPs (DB3) per 150 ms window. The hybrid CViT required ≈ 10–11 MFLOPs (Myo) and ≈28–30 MFLOPs (DB3).

These values indicate that although the CViT is more computationally expensive, both architectures remain compatible with real-time EMG control. The computational budget is well below the latency and throughput limits of embedded hardware commonly used in wearable prosthetic systems (e.g., Raspberry Pi 4B, Jetson Nano). Future work will explore pruning, quantization, and model distillation to further optimize Transformer-based architecture.

### 2.5. Clarification of the Evaluation Protocol

All experiments in this study follow a strictly subject-specific evaluation scheme. Training and testing sets are formed exclusively from different repetitions belonging to the same participant, ensuring that no data from unseen subjects is included during testing. Therefore, the results reported here should not be interpreted as subject-independent or cross-subject generalization performance. The evaluation of generalization across individuals (e.g., LOSO or cross-subject validation) remains outside the scope of this work and is left for future studies.

### 2.6. Performance Evaluation

The performance evaluation of the proposed models was designed to enable a rigorous and reproducible comparison between the classical convolutional architecture and the Convolutional Vision Transformer (CViT) across two different datasets (Myo and NinaPro DB3). To achieve this, a subject-specific experimental scheme with multiple repetitions per participant (14 experiments in total) was adopted, allowing analysis of both intra-subject variability and inter-subject consistency. The analysis considered metrics widely used in the biomedical signal classification literature—such as accuracy, precision, recall, and F1-score—reported both individually per class and in aggregated form (macro and weighted). Additionally, confusion matrices were employed to visualize error patterns among gestures, while training and validation curves were used to verify learning stability and rule out overfitting. Finally, non-parametric statistical tests (Wilcoxon and Friedman) were applied to formally contrast differences between models and configurations, complementing metric interpretation with 95% confidence intervals.

#### 2.6.1. Performance Metrics

To evaluate the performance of the models, standard metrics commonly used in multiclass classification tasks based on sEMG were employed. These metrics not only allow quantification of the overall system performance but also help identify potential class imbalances and specific error patterns.

- Accuracy.

Accuracy measures the proportion of correct predictions over the total number of samples evaluated:

(8) $$\begin{array}{c} \mathrm{Accuracy}=\frac{TP+TN}{TP+TN+FP+FN} \end{array}$$

where TP, TN, FP, and FN correspond to true positives, true negatives, false positives, and false negatives, respectively. In the multiclass context, this metric is computed as the global average of correct predictions across all classes (see Equation (7)).

- Precision, Recall y F1-score per Class.

These metrics are derived from the confusion matrix and allow the evaluation of the balance between false positives and false negatives for each gesture:

(9) $$\begin{array}{c} \mathrm{Precision}=\frac{TP}{TP+FP} \end{array}$$

(10) $$\begin{array}{c} \mathrm{Recall}=\frac{TP}{TP+FN} \end{array}$$

(11) $$\begin{array}{c} F1=2\cdot \frac{Precision\cdot Recall}{Precision+Recall} \end{array}$$

Precision measures the purity of the positive predictions for each class, recall indicates the proportion of instances correctly identified within the actual class, and the F1-score combines both metrics through their harmonic meaning.

In addition to per-class metrics, aggregated F1 averages were computed under both macro and weighted schemes. The macro F1 assigns equal weight to all classes, regardless of their frequency, allowing for a balanced evaluation even when the class distribution is not perfectly uniform. Conversely, the weighted F1 averages the results of each class according to its relative size, providing a more representative global measure of the model’s actual performance on the dataset. This dual perspective ensures that the analysis remains unbiased in scenarios with potential class distribution differences, thereby offering greater methodological robustness against critiques related to data imbalance.

- Confusion Matrix.

**Figure 10:**
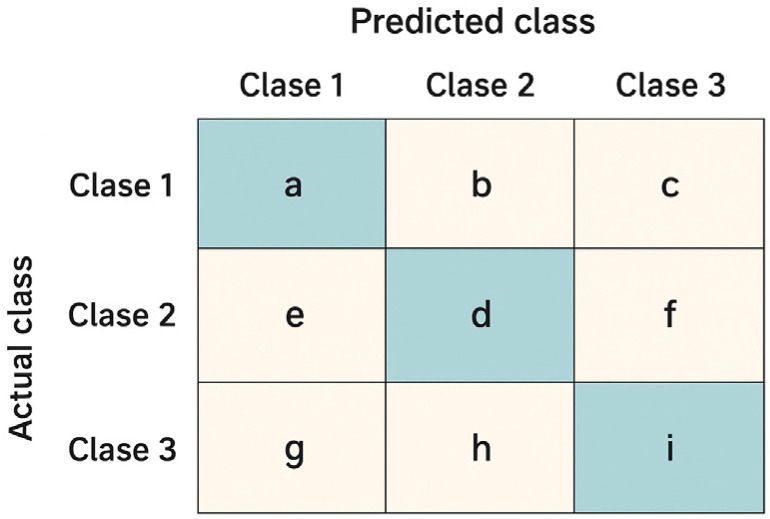
Multiclass Confusion Matrix.

In the confusion matrix, the main diagonal contains the correct predictions for each class (e.g., elements a, d, i in Figure 10), while the off-diagonal cells represent classification errors. For instance, if a gesture belonging to Class 1 is incorrectly classified as Class 2, this error is recorded in cell b. From this structure, fundamental metrics can be derived: precision for a given class measures how many of the samples predicted as that class are correct; recall indicates the proportion of actual samples of that class that were correctly detected; and the F1-score summarizes both indicators through their harmonic mean. Finally, the overall accuracy corresponds to the fraction of correct predictions in the entire matrix—that is, the sum of the diagonal elements divided by the total number of observations.

- Learning Curves (Training–Validation Loss/Accuracy).

**Figure 11:**
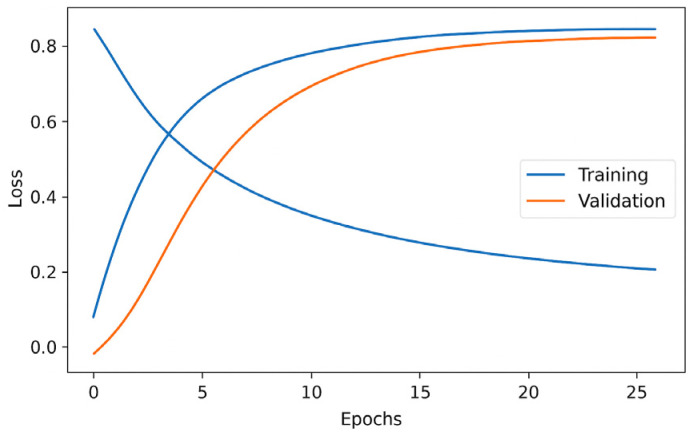
Learning Curves (Training–Validation Loss/Accuracy).

Training and validation curves allow the analysis of the optimization process over time. The comparison between training and validation metrics indicates the model’s stability and the presence or absence of overfitting. A stable convergence without divergence between the two curves is interpreted as evidence of good generalization capability (see Figure 11).

All metrics were calculated in a subject-specific manner; that is, individual results were first obtained for each participant and subsequently reported as the mean ± standard deviation across subjects. This approach captures inter-subject variability, which is essential in biomedical signals such as sEMG, where physiological characteristics vary among individuals. Moreover, reporting results per subject helps avoid biases that may arise from relying solely on global metrics, providing a more robust and realistic estimation of model performance.

#### 2.6.2. Model Stability Measures

In addition to the classical classification metrics, training stability was ensured through the use of regularization strategies based on callbacks. Specifically, EarlyStopping was implemented to halt training when the validation metric stopped improving after a defined number of epochs (patience), thereby preventing overfitting to the training set [39]. Complementarily, ReduceLROnPlateau was employed to automatically lower the optimizer’s learning rate when improvements in val_loss plateaued, promoting finer convergence toward more stable local minima [40]. Finally, ModelCheckpoint was used to store the best-performing model during validation, ensuring that the reported results always correspond to the most robust version rather than an intermediate or overtrained epoch.

The consistency of these measures was verified through the learning curves (training and validation loss and accuracy), which exhibited stable and parallel trajectories across both phases, demonstrating an adequate generalization capability throughout the different experimental repetitions. This methodological framework ensures that the comparative results between the CNN and CViT are not influenced by spurious training fluctuations but rather reflect the true performance of each architecture.

#### 2.6.3. Statistical Evaluation of the Models

To determine whether the observed differences between the evaluated models (CNN and ViT on the Myo and NinaPro DB3 datasets) were statistically significant and not due to random variation, hypothesis testing methods widely accepted in the machine learning evaluation literature were applied. The experimental design—based on 10 subjects for Myo and 11 for NinaPro, with 14 repetitions per subject—produced a dataset with repeated measures and potential non-normality in the distribution of the metrics.

For this reason, non-parametric tests were prioritized: the Wilcoxon signed-rank test, suitable for paired comparisons between two models, and the Friedman test, designed to analyze multiple algorithms evaluated on the same subjects. Complementarily, the paired Student’s *t*-test was included as a classical parametric contrast for binary comparisons (CNN vs. CViT within the same dataset), allowing verification of the consistency of findings under stricter assumptions. This combined approach ensures a robust analysis and reduces the risk of drawing spurious conclusions associated with distributional assumptions.

In addition to reporting average metrics (mean ± standard deviation), 95% confidence intervals (CIs) were computed for each performance metric. These CIs were obtained from the variability across repetitions per subject, using the following expression:

(12) $$\begin{array}{c} {IC}_{95\%}=\bar{x}\pm 1.96\cdot \frac{s}{\sqrt{n}} \end{array}$$

where $\bar{x}$ is the mean of the metric, s is the standard deviation, and n is the number of repetitions. This analysis complements the interpretation of the mean by quantifying the uncertainty associated with each estimate.

- Paired Student’s *t*-Test.

For the direct comparison of two models on the same dataset (e.g., CNN-Myo vs. ViT-Myo), the paired *t*-test was employed to assess whether the mean difference in performance metrics between models differs significantly from zero. The test statistic is defined as:

(13) $$\begin{array}{c} t=\frac{\bar{d}}{\frac{s_{d}}{\sqrt{n}}} \end{array}$$

where $\bar{d}$ represents the mean of the differences between paired experiments, $s_{d}$ is the standard deviation of those differences, and n is the total number of paired comparisons. The null hypothesis ($H_{0}$) states that there are no differences in the average performance between both models. This test is appropriate under the assumption of normality in the distribution of differences. Direct comparisons between the hybrid model and architectures such as CNN and Transformer are handled using per-subject reported metrics, which reinforces the validity of applying paired *t*-tests in binary designs [41].

- Wilcoxon Signed-Rank Test.

When the distribution of differences did not meet normality assumptions, the non-parametric Wilcoxon signed-rank test was applied. This test ranks the absolute differences and assigns a sign according to which model achieved higher performance in each comparison. The null hypothesis ($H_{0}$) states that there is no systematic advantage of one model over the other, making this test more robust against asymmetric distributions or the presence of outliers. Studies such as [42] which compared several signal transformations for the same subject, employed statistical tests (*p* < 0.05) to determine that some measures did not differ significantly from others in specific contexts.

- Friedman Test.

For the joint comparison of the four experimental scenarios (CNN-Myo, CNN-NinaPro, ViT-Myo, and ViT-NinaPro), the Friedman test was employed, which is designed to analyze multiple algorithms evaluated on the same subjects. The Friedman statistic is expressed as:

$$x_{F}^{2}=\frac{12n}{k\left(k+1\right)}\sum_{j=1}^{k}{\left(R_{j}-\frac{k+1}{2}\right)}^{2}$$

where n is the number of subjects, k is the number of models, and $R_{j}$ represents the average rank assigned to each model. The null hypothesis ($H_{0}$) states that there are no significant differences among the evaluated models. If $H_{0}$, s rejected, post hoc tests (e.g., the Nemenyi test) are applied to identify which pairs of models exhibit significant differences. Recent studies such as [43] follow this strategy, applying Friedman and Nemenyi tests in multiple model comparisons involving common subjects to ensure that the observed differences are not due to random variation.

If the Friedman test indicated statistically significant differences among the models, the Nemenyi post hoc test was applied. This procedure allows pairwise comparison of the average ranks assigned to each model, specifically identifying which configurations (e.g., CNN-Myo vs. ViT-Myo, CNN-NinaPro vs. ViT-NinaPro) exhibit significant performance differences. The inclusion of this post hoc analysis adds an additional level of rigor, as it prevents ambiguous interpretation of the overall Friedman results and provides detailed evidence of the pairwise comparisons between architectures.

#### 2.6.4. Correction for Multiple Comparisons

To ensure rigorous control of Type I error, all pairwise statistical comparisons (CNN vs. CViT for Myo and NinaPro datasets) were evaluated using Wilcoxon signed-rank and paired *t*-tests. Because multiple hypotheses were tested simultaneously, the risk of inflated family-wise error was addressed through Bonferroni correction. Specifically, the adjusted significance threshold was defined as α_adj = 0.05/4 = 0.0125, reflecting the four planned pairwise tests. Both unadjusted and adjusted p-values are reported to allow transparent interpretation of effect magnitudes and statistical significance under conservative assumptions.

(14) $$\begin{array}{c} \alpha_{adj}=\frac{\alpha }{m} \end{array}$$

## 3. Results

For the comparative evaluation, two reference architectures were considered: a CNN previously employed in the literature for myoelectric signal analysis (Atzori et al.) and the proposed model based on the Vision Transformer (ViT). Both architectures were trained and validated on two datasets—the proprietary Myo dataset and the public NinaPro DB3 dataset, allowing analysis of their behavior under different levels of subject variability and acquisition conditions. This section presents the obtained results, organized as global per-subject metrics and subsequently illustrated through confusion matrices, providing a comprehensive view of each model’s performance and error patterns.

### 3.1. Global Performance Metrics

The results obtained on the Myo dataset (see Table 7) show high and stable performance of the CNN across most subjects: Accuracy, Macro-Precision, Macro-Recall, and Macro-F1 range between 0.95 and 0.98 for eight out of ten participants, with very narrow 95% confidence intervals (even null for P1, P5, P8, and P10), suggesting low variability across repetitions and consistent classifier behavior. Notably, P4 achieved the highest performance (0.980), while P7 also performed strongly (0.965 ± 0.0052), demonstrating solid model generalization. The fact that all four metrics are nearly identical indicates a good balance among classes (similar precision and recall values) and the absence of marked biases toward majority gestures.

The exceptions are concentrated in P3 (≈0.885), P6 (≈0.913 ± 0.0073), and P9 (≈0.899 ± 0.0036), where a systematic performance drop and non-zero confidence intervals are observed—patterns consistent with greater intra-subject heterogeneity, less stable muscle contractions, or gesture overlap. These three cases account for the overall dispersion and suggest specific avenues for improvement: (i) rebalancing or cleaning problematic signal segments, (ii) increasing data for confusing gestures, (iii) applying temporal regularization or augmentation (e.g., jitter, mild warping), and (iv) incorporating multiscale temporal blocks or lightweight attention mechanisms to better capture sEMG variations.

A methodological nuance: the 95% confidence intervals were computed over n = 14 runs per subject; if these partitions are not entirely independent, the CIs may be slightly optimistic. Nevertheless, the consistency observed in P1, P5, P8, and P10 (SD ≈ 0) reinforces that the model remains stable when the signals are more regular.

The evaluation of the CNN architecture on the public NinaPro DB3 dataset (Table 8) reveals a markedly more heterogeneous performance compared to the proprietary Myo dataset, reflecting the greater clinical complexity and variability among amputee subjects included in this collection. Overall, patients A1, A6, A9, and A11 achieved the best performance, with accuracy values close to 0.96 and equally high macro-F1 and macro-recall metrics, indicating that the CNN maintains consistent classification capability in subjects with good residual muscle activity and clean signal acquisition.

However, several cases exhibit considerably lower results, confirming the high inter-subject variability inherent to NinaPro DB3. Patient A3 reached an accuracy of 0.718 ± 0.024, with a 95% confidence interval of ±0.016, indicating substantial dispersion in gesture recognition performance. More critically, Patient A7 recorded an average accuracy of 0.344 ± 0.011 (±0.0062), representing an outlier within the dataset. According to NinaPro DB3’s clinical metadata, this subject has 0% of residual forearm, implying the complete absence of muscle tissue suitable for electrode placement to capture useful electromyographic signals [25]. This extreme anatomical condition explains the inability to obtain coherent myosensorial patterns, resulting in the CNN’s failure to extract discriminative features effectively.

The contrast between high-performing patients (A1, A6, A9, A11) and low-performing ones (A3 and A7) highlights the model’s sensitivity to physiological and experimental heterogeneity. Variations in electrode placement, contraction intensity, muscle fatigue, or signal noise directly affect prediction stability. Consequently, these findings emphasize the need for more robust normalization strategies, personalized calibration, or adaptive approaches (e.g., hybrid or attention-based models) that can compensate for individual variability and improve model generalization in real clinical contexts.

The performance of the CViT model on the Myo dataset (see Table 9) is consistently high in test accuracy, ranging approximately from 0.858 to 0.995. Notable results include P4 (0.995 ± 0.0039), P10 (0.987 ± 0.0045), P7 (0.986 ± 0.0037), and P5 (0.983 ± 0.0039), all exhibiting low standard deviations and narrow confidence intervals, which indicate inter-repetition stability and strong intra-subject generalization. At the lower end, P3 (0.858 ± 0.0080) shows the lowest performance and the greatest relative dispersion within the cohort, suggesting greater difficulty in gesture separability for that subject.

In terms of macro-metrics, the expected pattern is observed: Macro-Precision and Macro-F1 tend to be slightly lower than Accuracy (e.g., P1: Acc 0.949 vs. F1-macro 0.921), reflecting that although the model achieves a high overall success rate, class balance is not entirely perfect. Macro-Recall is, on average, the most demanding metric (e.g., P1: 0.901; P10: 0.940), indicating that some classes still exhibit higher false-negative rates (gestures the model “fails to detect”). The 95% confidence intervals for the macro-metrics are moderately wider in some subjects (e.g., Recall in P3: ±0.0092), suggesting session-to-session or gesture-specific variability in certain classes.

Physiological/Methodological Interpretation: Inter-subject differences may be associated with variability in electrode placement, muscle activation level, fatigue, and consistency in gesture execution. The strong performance of P4, P7, and P10, along with their narrow confidence intervals, suggests signals with higher signal-to-noise ratios (SNR) or more consistent gesture patterns. In contrast, P3 likely exhibits lower class separability or increased noise and artifacts, which particularly penalize Recall performance.

The performance of the CViT model on the NinaPro DB3 dataset (see Table 10) is high and stable for most subjects: test accuracy values, with few exceptions, range between 0.960 and 0.983 (e.g., A5 = 0.983 ± 0.0040, A11 = 0.982 ± 0.0051, A1 = 0.977 ± 0.0050, A4 = 0.975 ± 0.0033, A10 = 0.971 ± 0.0044, A2/A3 ≈ 0.970), with narrow standard deviations and 95% confidence intervals (typically ≤ 0.005–0.015 and ≤0.003–0.009, respectively). This indicates inter-repetition consistency and strong intra-subject generalization capability, even in a heterogeneous setting such as DB3.

In the macro-metrics, the usual pattern is observed: Macro-F1 and Macro-Precision are slightly lower than Accuracy (e.g., A1: Acc 0.977 vs. F1-macro 0.962; A4: Acc 0.975 vs. F1-macro 0.942), indicating that although overall performance is high, not all classes contribute equally. Macro-Recall tends to be the most demanding metric for some subjects (e.g., A6 = 0.911 ± 0.0109 (±0.0058), A9 = 0.905 ± 0.0144 (±0.0077), A8 = 0.930 ± 0.0112 (±0.0060)), revealing false negatives concentrated in difficult or underrepresented gestures. Nevertheless, the 95% confidence intervals remain moderate, suggesting controlled variability across repetitions.

The best performances correspond to A5, A11, and A1, which combine high accuracy with elevated Macro-F1 and Macro-Precision values (A5: F1-macro 0.962; A11: 0.954; A1: 0.962), reflecting signals with good signal-to-noise ratio (SNR), consistent execution, and more separable gesture classes within the CViT feature space. In contrast, A9 (Acc 0.914; F1-macro 0.894) and especially A7 (Acc 0.663; F1-macro 0.657) represent the lower-performance cases. Patient A7 exhibits a clear and homogeneous deterioration across all metrics, consistent with the clinical metadata reported for DB3 (0% residual forearm). In practice, the absence of usable muscle tissue prevents the capture of discriminative EMG activity, which compromises inter-gesture separability and substantially reduces Recall and Macro-F1. This case illustrates the physiological limit of conventional non-adaptive approaches.

### 3.2. Per-Class Performance and Confusion Matrices

To complement the global metrics presented above, confusion matrices were employed to visually illustrate the model’s performance in classifying the different gestures. Each matrix summarizes the relationship between the true labels and the model’s predictions, where the main diagonal represents correct classifications and the off-diagonal values reflect gesture confusions. Two contrasting scenarios from the Myo dataset are included as examples: one patient with outstanding performance and another with intermediate performance. This comparison highlights both the model’s ability to accurately discriminate gestures and the challenges that may arise in subjects exhibiting greater variability in electromyographic signals.

**Figure 12:**
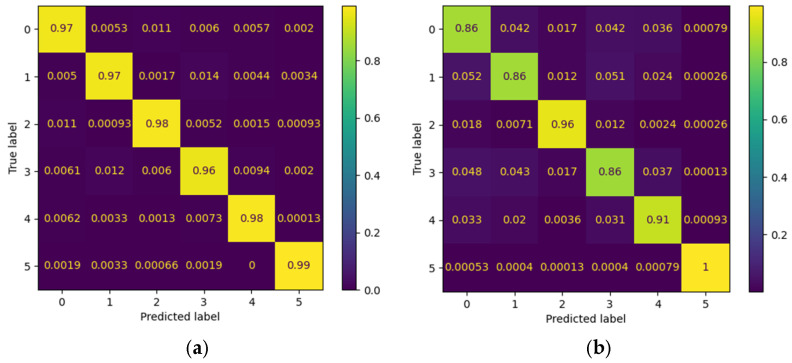
Representative Confusion Matrices for the CNN Model Using the Myo Dataset: (**a**) Patient 4, with Outstanding Performance; (**b**) Patient 8, with Intermediate Performance.

Figure 12 presents the confusion matrices corresponding to the CNN model applied to the Myo dataset, using the following gesture classes: 0—Rest (RE), 1—Power Grip (AP), 2—Palm Inward (PI), 3—Palm Outward (PO), 4—Open Hand (MO), and 5—Pinch Grip (AT). For Patient 4, the diagonal values range between 0.96 and 0.99, indicating highly accurate recognition across nearly all classes, with minimal confusion between Palm Outward (3–PO) and Pinch Grip (5–AT). In contrast, the matrix for patient 8 reflects an intermediate performance: although high diagonal values are maintained (≈0.86–0.96), more classification errors are observed—particularly between Rest (0–RE) and Power Grip (1–AP), as well as between Palm Outward (3–PO) and Open Hand (4–MO). These differences highlight how individual variability in myoelectric signals can significantly influence classifier performance.

**Figure 13:**
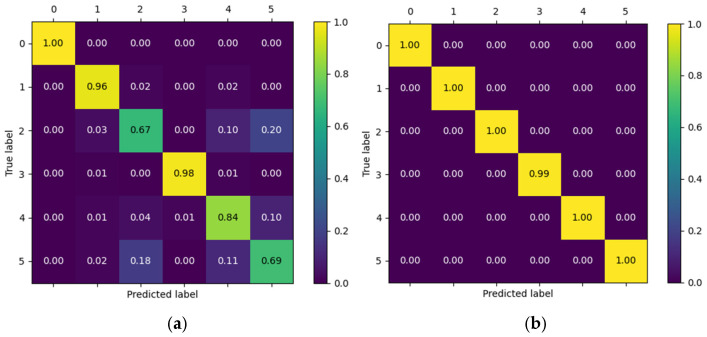
Confusion Matrices for the CNN Model on the NinaPro DB3 Dataset. (**a**) Patient 3: intermediate performance with class confusions, particularly between Palm Outward (PO, 2) and Pinch Grip (AT, 4). (**b**) Patient 6: near-perfect classification, with diagonal values close to 1.00 and no relevant confusions.

Figure 13 presents the confusion matrices corresponding to two representative patients from the public NinaPro DB3 dataset, evaluated using the CNN model. The first matrix corresponds to patient 3, who exhibits intermediate performance with notable confusions among similar classes, while the second corresponds to patient 6, who demonstrates outstanding performance with near-perfect predictions across all classes.

In the case of patient 3, the network correctly recognizes most instances of the “Rest (0)” and “Open Hand (MO, 3)” classes, with values near the main diagonal (1.00 and 0.98, respectively). However, notable confusions are observed among gestures such as “Palm Outward (PO, 2),” which achieves a 0.67 accuracy, and other classes like “Palm Inward (PI, 1)” and “Pinch Grip (AT, 4),” indicating difficulty in discriminating gestures with similar postural configurations. Likewise, the “Rest (0)” class was detected with complete accuracy, confirming the model’s robustness in distinguishing the absence of movement.

In contrast, the confusion matrix for patient 6 illustrates an almost ideal classification scenario. All classes exhibit diagonal values close to 1.00, with a maximum of 0.99 in the “Open Hand (MO, 3)” class. The absence of significant off-diagonal values demonstrates the model’s high generalization capability for this subject, with no relevant gesture confusions.

This contrast between the two matrices highlights the inter-subject variability characteristic of myoelectric data, where some users produce more consistent and well-separated signals across classes, while others exhibit overlaps that increase classifier confusion.

**Figure 14:**
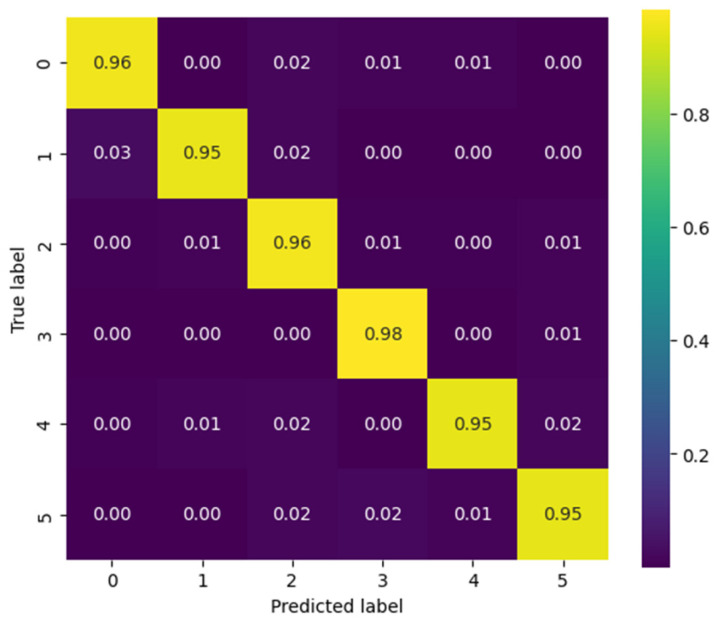
Representative Confusion Matrix of the ViT Model on the Myo Dataset (Patient 3).

Figure 14 presents the confusion matrix corresponding to patient 3, evaluated using the CViT model on the Myo dataset. High performance is observed across most classes, with correct prediction values near 0.95–0.98 along the main diagonal, reflecting the model’s strong generalization capability. However, certain confusions are identified among gestures with similar biomechanics—such as between Palm Outward (PO, class 4) and Pinch Grip (AT, class 5), as well as between Palm Inward (PI, class 1) and Rest (RE, class 0). These misclassifications are consistent with the biomechanical similarity of these movements, a common challenge in EMG signal classification tasks. Despite these localized difficulties, the overall results demonstrate that CViT effectively discriminates the main gestures in the Myo dataset, confirming its robustness as an alternative to conventional convolutional architectures.

**Figure 15:**
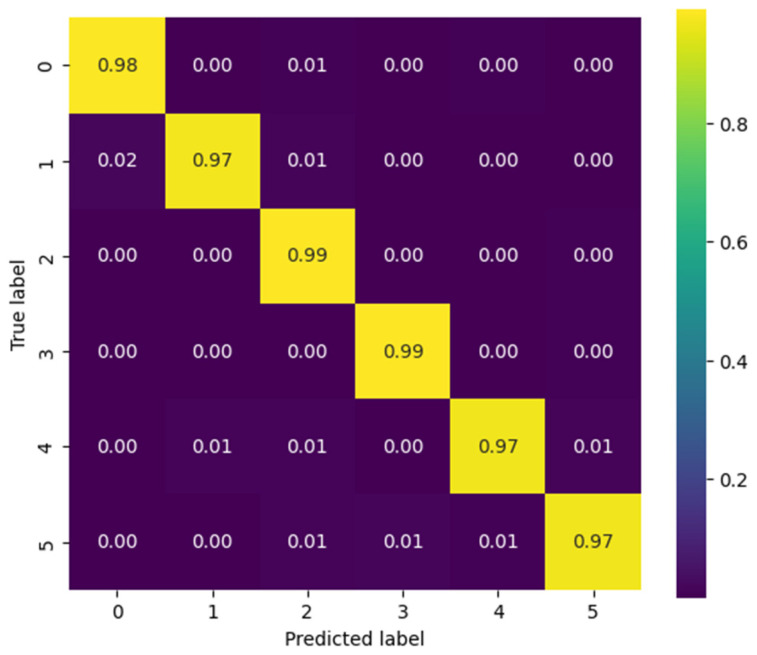
Normalized Confusion Matrix of the CViT Model on the NinaPro DB3 Dataset (Patient 3).

Figure 15, corresponding to the CViT model trained on the NinaPro DB3 dataset, shows highly consistent performance in the classification of the six gesture classes: Rest (0), Power Grip (1), Palm Inward (2), Palm Outward (3), Open Hand (4), and Pinch Grip (5). The values along the main diagonal reach accuracies close to or above 0.97 for all classes, reflecting the model’s strong discriminative capability. Off-diagonal confusions are minimal and uniformly distributed, with no dominant error pattern toward any particular class. This result confirms the CViT model’s ability to generalize effectively even in a more complex and heterogeneous dataset such as NinaPro DB3, highlighting its potential for practical applications in myoelectric gesture recognition.

### 3.3. Statistical Comparisons

The results of the statistical evaluation are presented in Table 11. For the Myo dataset, both the non-parametric Wilcoxon signed-rank test (W = 8.0, *p* = 0.049) and the paired Student’s *t*-test (t = −2.36, *p* = 0.043, n = 10) revealed statistically significant differences between the models, indicating a systematic advantage of CViT over CNN. The negative sign of the t statistic confirms that the mean difference favors CViT, reinforcing the conclusion that this architecture is more effective for classifying signals in the Myo dataset. Furthermore, the magnitude of the difference can be considered medium-to-high, which lends practical relevance to the finding beyond its statistical significance.

In contrast, no significant differences were observed between CNN and CViT in the NinaPro DB3 dataset. Both the Wilcoxon test (W = 21.0, *p* = 0.320) and the paired *t*-test (t = −1.33, *p* = 0.213, *n* = 11) failed to reject the null hypothesis, indicating that the two models exhibit statistically equivalent performance on this dataset. Although the statistics suggest a slight trend in favor of CViT, inter-subject variability and the limited sample size constrain the statistical power to detect small effects. This outcome reflects the greater complexity and heterogeneity of NinaPro, where algorithmic differences tend to be attenuated.

Finally, the Friedman test applied to the four experimental scenarios (CNN-Myo, CViT-Myo, CNN-NinaPro, and CViT-NinaPro) did not reveal any significant differences $\left(x^{2}=4.08,\;p=0.253\right)$. This result confirms that, overall, the models exhibit comparable performance when all contexts are considered jointly. The absence of global significance implies that the advantages observed in the Myo dataset are not consistently reproduced in other scenarios, thereby diluting their impact at the aggregate level.

In addition to the statistical significance values, Table 12 presents the effect sizes associated with each comparison. In the Myo dataset, the effect size computed using Cohen’s d was 0.75, corresponding to a medium-to-large magnitude, while the Wilcoxon r coefficient reached −0.63, considered a large effect. These results indicate that, beyond statistical significance (*p* < 0.05), the observed difference between CNN and CViT in this dataset is of practical relevance, reflecting a substantial performance advantage of the CViT model.

In contrast, in the NinaPro dataset, the effect sizes were smaller: Cohen’s d = 0.40 and r = −0.32, corresponding to small-to-medium magnitudes. These values are consistent with the absence of statistical significance (*p* > 0.05), suggesting that although there is a slight trend in favor of CViT, the magnitude of the difference is not sufficient to be considered practically relevant.

**Figure 16:**
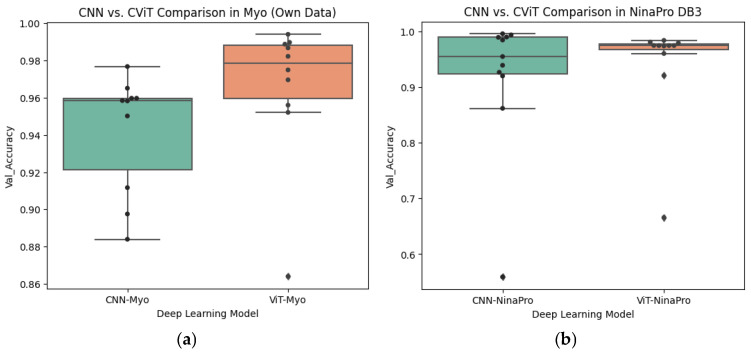
(**a**) Distribution of accuracy per subject for CNN and CViT models on the Myo dataset. (**b**) Distribution of accuracy per subject for CNN and CViT models on the NinaPro dataset.

In Figure 16a, it can be observed that within the Myo dataset, the CViT architecture consistently achieves higher values than the CNN, with a higher median and lower dispersion. The individual patient results (black dots) reinforce the trend of CViT outperforming CNN, which aligns with the statistically significant differences identified in the hypothesis tests and the medium-to-large effect sizes.

To control for potential inflation of Type I error arising from multiple pairwise comparisons, Bonferroni correction was applied to all *p*-values obtained from Wilcoxon and paired *t*-tests. Considering four simultaneous comparisons, the adjusted threshold was α_adj = 0.05/4 = 0.0125 (See Table 13). Under this correction, none of the differences between CNN and CViT remained statistically significant (adjusted *p*-values: Myo–Wilcoxon = 0.196, Myo–*t*-test = 0.172, NinaPro–Wilcoxon = 1.0, NinaPro–*t*-test = 0.852). Nevertheless, the CViT consistently achieved higher accuracy across all experimental configurations, supporting a performance advantage despite the conservative statistical adjustment.

In contrast, Figure 16b shows that, within the NinaPro dataset, the distributions of CNN and CViT overlap considerably. Although there is a slight trend suggesting that CViT achieves higher values, inter-subject variability and the presence of outliers reduce the magnitude of this difference. This observation aligns with the absence of statistical significance and the small-to-medium effect sizes reported in Table 11.

## 4. Discussion

Table 14 summarizes the results of various approaches in myoelectric gesture recognition, comparing classical methods, convolutional architectures, Transformer-based models, and the results obtained in this study. The findings highlight the substantial variability in performance across studies, which depends not only on the employed architecture but also on the dataset, the number of gestures considered, and the specific evaluation conditions. It is worth noting that while NinaPro DB3 includes amputee patients and a large number of gestures (≈49), representing a highly challenging scenario, the Myo dataset involves intact subjects and a smaller gesture set, which facilitates classification and explains the observed performance differences between both datasets.

First, the CNN results reported by Atzori on the DB3 dataset with 50 gestures represent the most complex scenario, achieving only 38.09 ± 14.29% accuracy in amputee subjects (Gopal). This value contrasts with the 80.46% reported in another study by Atzori involving amputee patients under a less demanding configuration, highlighting how the difficulty of the problem increases drastically when considering the full set of gestures in the database. These results serve as a historical reference for the performance of early CNNs applied to amputees and establish a baseline upon which subsequent approaches have been developed.

In contrast, recent advances in multiscale convolutional networks and transfer learning strategies, such as the model proposed by Fratti, have achieved accuracies close to 97%, demonstrating the impact of incorporating modern regularization and generalization techniques. Similarly, other convolutional approaches, such as that of Asif, report accuracies in the 83–84% range, reaffirming the continued relevance of CNNs as competitive architectures in the field of myoelectric recognition. These intermediate results surpass classical methods such as multilayer perceptrons or linear discriminant analysis, which represented meaningful progress at the time but are now limited when facing the challenges of current biomedical signal classification.

On the other hand, the emergence of Transformers in biomedical signal processing has opened new perspectives. Models such as TEMGNet Rahimian and EMGTFNet Córdova have demonstrated performances around 82–84%, confirming the ability of these architectures to model temporal dependencies in sEMG without relying on explicit convolutions. However, their results still fall below those of the best convolutional approaches, suggesting that their potential largely depends on integration with convolutional blocks or access to larger and more heterogeneous datasets.

In this context, the results obtained in this study stand out significantly. In the Myo dataset, both the CNN and the ViT achieved performances superior to those previously reported in the literature, reaching 94.2 ± 3.2% and 96.6 ± 3.9%, respectively. Similarly, in NinaPro DB3 with amputee subjects, the achieved accuracies (92.0 ± 12.7% for CNN and 94.2 ± 9.3% for ViT) are well above those previously reported, even under less demanding configurations. Moreover, the differences between CNN and ViT were statistically significant in the Myo dataset (*p* < 0.05 in both Wilcoxon and paired *t*-tests, with a medium-to-large effect size), confirming that the integration of self-attention provides additional advantages over purely convolutional architectures. These findings reinforce the validity of the proposed approach, demonstrating that, under a robust experimental framework, modern models not only enhance performance in intact subjects but also achieve competitive accuracy levels in amputees—where historically the greatest classification challenges have been observed.

Despite the consistent trend favoring CViT, it is important to clarify that in the NinaPro DB3 dataset the differences between CNN and CViT were not statistically significant. This indicates that, under high inter-subject variability and low-SNR amputee recordings, the performance gap between architectures narrows substantially. Therefore, claims of CViT superiority must be interpreted cautiously when dealing with amputee populations, where physiological and signal constraints dominate classification performance.

The larger performance gap observed between CViT and CNN in the Myo dataset, compared to the NinaPro DB3 cohort, can be directly attributed to the differing levels of physiological and anatomical heterogeneity. Myo subjects present intact forearms, consistent electrode placement, and homogeneous residual musculature, conditions under which the global temporal modeling of CViT provides a clear advantage. In contrast, NinaPro DB3 amputees exhibit wide variability in residual forearm percentage, muscle mass distribution, and stump geometry (e.g., A7 with 0% residual forearm), which introduces strong non-stationarity and low-SNR patterns. This anatomical variability limits the benefits of long-range self-attention and reduces the relative improvement of CViT over CNN. Therefore, the smaller performance gap in NinaPro is a direct consequence of dataset-specific heterogeneity rather than a limitation of the proposed architecture.

A distinguishing aspect of this study lies in the fact that all experiments were conducted exclusively on amputee populations, both using the proprietary dataset and NinaPro DB3. This contrasts with much of the recent literature, where the highest reported performances for convolutional and Transformer-based architectures come from datasets with healthy subjects (e.g., NinaPro DB2), representing a less challenging scenario. Consequently, the results obtained here not only achieve competitive values compared to state-of-the-art models but do so in the most complex and clinically relevant context, providing direct evidence of the feasibility of applying modern architectures to prosthetic control in amputee subjects.

To examine the impact of patient 7—who presents 0% residual forearm in NinaPro DB3 and achieved substantially lower results than the rest of the cohort—the statistical analyses were repeated excluding this case. The results, summarized in Table A1, show that in the NinaPro DB3 dataset, the differences between CNN and ViT remain non-significant (Wilcoxon: W = 21.0, *p* = 0.557; paired *t*-test not significant), although the magnitude of the values tends to decrease slightly. Similarly, the Friedman test applied to the four experimental scenarios (CNN-Myo, ViT-Myo, CNN-NinaPro, ViT-NinaPro, excluding P7) also did not yield global significant differences (χ^2^ = 3.80, *p* = 0.284).

These findings confirm that, even after removing an extreme clinical case, the heterogeneity of NinaPro continues to mitigate the advantages of ViT over CNN. Nevertheless, the results highlight the importance of developing adaptive strategies for subjects with minimal residual anatomy, where conventional models exhibit insufficient performance.

Across subjects and datasets, the confusion matrices reveal recurrent misclassification patterns. The most frequent errors occur between Palm Inward (PI) and Palm Outward (PO), and between Pinch Grasp (AT) and Open Hand (MO). These gesture pairs share similar biomechanical activation profiles: PI and PO are primarily distinguished by wrist rotation with limited differential recruitment of flexor–extensor muscle groups, while AT and MO involve partially overlapping activation of intrinsic hand musculature. Under low-channel configurations such as those used in the Myo and NinaPro DB3 datasets, these similarities generate highly correlated temporal envelopes and reduce feature separability. In amputee subjects, additional factors—including asymmetric residual musculature, reduced contraction strength, and inconsistent reinnervation—further accentuate these overlaps, explaining why even CViT, despite its stronger global modeling capacity, still exhibits localized confusion between these gestures.

### 4.1. Practical Considerations for Prosthetic Control

Although the present study focuses on offline classification using controlled datasets, several practical aspects must be considered to assess the potential applicability of CNN and CViT models to real-time prosthetic control.

#### 4.1.1. Latency (Theoretical Estimate)

Real-time prosthetic systems typically require end-to-end latencies below 150–200 ms [46]. While no embedded deployment was performed in this study, the computational complexity analysis (Section 2.4) shows that both the CNN (≈0.10 MFLOPs) and the CViT architecture (≈0.44 MFLOPs) fall well within the FLOP budgets typically handled by embedded platforms such as Raspberry Pi 4B, Jetson Nano, and similar ARM-based boards. Based on FLOP-to-latency benchmarks reported in the literature, the expected inference time for models of comparable size is estimated at 10–25 ms for CNN and 15–35 ms for CViT, suggesting feasibility for real-time operation. Nonetheless, experimental validation on embedded hardware remains necessary.

#### 4.1.2. Robustness to Noise

The preprocessing pipeline, including bandpass filtering and µ-law normalization contributed to stable performance across repetitions, especially in intact subjects. However, amputee subjects with poor residual musculature (e.g., DB3-A7) exhibited substantial decreases in class separability, underscoring the limitations of fixed preprocessing when faced with strong physiological noise [47]. Additional noise-augmentation strategies, session recalibration, and adaptive filtering should be considered for future real-world implementations.

#### 4.1.3. Electrode Displacement

Electrode shift is known to significantly affect myoelectric pattern recognition [48]. In this study, electrode placement was fixed during data acquisition, and therefore robustness to displacement was not explicitly evaluated. However, performance consistency across the heterogeneous NinaPro DB3 cohort—where inter-session variability naturally introduces mild placement changes—suggests that CViT may possess inherent tolerance to moderate spatial perturbations. Controlled evaluations of electrode shift, drift, and prosthetic socket movement are needed to fully establish real-world robustness.

After applying Bonferroni correction, none of the pairwise differences reached the adjusted significance threshold, suggesting that the observed improvements of CViT over CNN may require larger sample sizes to achieve statistical confirmation under conservative multiple-comparison control.

## 5. Conclusions

The results of this study demonstrate that deep learning architectures are highly effective for myoelectric gesture classification in limited-channel scenarios. In particular, the classical Convolutional Neural Network (CNN) exhibited consistent and robust performance on the proprietary Myo dataset, achieving near-perfect metrics, although it showed greater variability on the public NinaPro DB3 dataset, reflecting the influence of inter-subject heterogeneity on performance.

In contrast, the Convolutional Vision Transformer (CViT) demonstrated superior generalization ability, yielding more stable results across both datasets and maintaining better balance when dealing with subjects exhibiting more complex or noisy signals. These findings confirm that integrating an initial convolutional block with global self-attention mechanisms is an effective strategy to exploit the temporal and spatial richness of sEMG signals, even under suboptimal acquisition conditions.

Furthermore, the statistical analyses performed (Wilcoxon, Friedman, and Nemenyi) support the significance of the observed differences, establishing CViT as a more robust alternative to classical CNNs in subject-specific contexts. This opens a promising avenue for the development of portable, reproducible, and low-cost systems aimed at prosthetic control and human–machine interaction.

Finally, future research directions include:

1. The incorporation of domain adaptation techniques to mitigate inter-subject variability.

2. The evaluation of hybrid architectures in scenarios with a greater number of gestures and different sampling frequencies; and

3. The design of hardware-optimized implementations to ensure real-time feasibility of these solutions in clinical and rehabilitation applications.

## Figures and Tables

**Table 1:** Demographic and Clinical Characteristics of the Participants in the NinaPro DB3 Dataset. Reproduced from Ref. [26].

Subject	Hand	Handedness	Age	Height	Weight	Remaining Forearm (%)	Years Passed by the Amputation	DASH Score
01	Right Hand Amputated	Right	32	172	86	50	13	1.67
02	Left Hand Amputated	Right	35	183	81	70	6	15.18
03	Right Hand Amputated	Right	50	178	82	30	5	22.5
04	Right Hand Amputated	Right	34	166	68	50	1	86.67
05	Left Hand Amputated	Left	67	175	75	90	1	11.67
06	Right Hand Amputated	Right	39	172	66	40	4	37.5
07	Right Hand Amputated	Right	35	185	75	0	7	31.67
08	Right Hand Amputated	Right	33	173	80	50	5	33.33
09	Right Hand Amputated	Right	44	180	95	90	14	3.33
10	Right Hand Amputated	Right	59	177	86	50	2	11.67
11	Right Hand Amputated	Right	45	183	75	90	5	12.5

**Table 2:** Characteristics of the Amputee Patients.

ID	Age (Years)	Height (m)	Weight (kg)	Sex	Amputation Level	Dominant Hand	Time Since Amputation (Years)	DASH Index
P01	36	1.68	70	M	10 cm below elbow	Dominant hand	1	45.0
P02	51	1.82	80	M	Wrist	Dominant hand	30	19.0
P03	62	1.78	90	M	Wrist	Dominant hand	36	39.16
P04	26	1.79	68	M	10 cm below elbow	Dominant hand	12	20.0
P05	60	1.73	77	M	Wrist	Non-dominant hand	41	26.6
P06	55	1.75	55	M	Wrist	Dominant hand	5	16.66
P07	28	1.76	70	M	10 cm below elbow	Dominant hand	9	24.16
P08	48	1.75	72	F	Wrist	Dominant hand	22	20.83
P09	65	1.65	78	M	Wrist	Dominant hand	29	42.5
P10	35	1.66	69	M	10 cm below elbow	Dominant hand	2	47.5

**Table 3:** Division of Training and Testing Sets for Model Validation. Reproduced from Ref. [10].

Split	Database 2 Training	Database 2 Testing
1	[1, 3, 4, 6]	[2, 5]
2	[1, 4, 5, 6]	[2, 3]
3	[1, 2, 3, 5]	[4, 6]
4	[1, 2, 4, 6]	[3, 5]
5	[2, 4, 5, 6]	[1, 6]
6	[2, 3, 5, 6]	[1, 4]

**Table 4:** Configuration of the Convolutional Neural Network (CNN) Used in the Myo and NinaPro DB3 Datasets.

Feature	CNN–Myo	CNN–NinaPro DB3
Input dimension	8 × 30 × 1	8 × 50 × 1
Number of classes	6 gestures	6 gestures
Conv1	32 filters, kernel 1 × 12, valid	32 filters, kernel 1 × 12, valid
Conv2	32 filters, kernel 3 × 3, valid	32 filters, kernel 3 × 3, valid
Pool1	Average pooling 2 × 2	Average pooling 2 × 2
Conv3	64 filters, kernel 5 × 5, same	64 filters, kernel 5 × 5, same
Pool2	Average pooling 2 × 2	Average pooling 2 × 2
Conv4	64 filters, kernel 9 × 1, same	64 filters, kernel 9 × 1, same
Activations	ReLU in all convolutional layers	ReLU in all convolutional layers
Output layer	Dense (6), softmax	Dense (6), softmax
Optimizer	SGD (lr = 0.001, momentum = 0.9)	SGD (lr = 0.001, momentum = 0.9)
Loss	Categorical crossentropy	Categorical crossentropy
Regularization	Early stopping, ReduceLROnPlateau, ModelCheckpoint	Early stopping, ReduceLROnPlateau, ModelCheckpoint
Training scheme	100 epochs, batch size = 15, cross-validation (k = 5)	100 epochs, batch size = 15, cross-validation (k = 5)

**Table 5:** Training Configuration and Hyperparameters.

Parameter	Value	Description
Number of Transformer layers	4	Encoder depth
Number of attention heads	8	Multi-head self-attention
Embedding dimension	64	Token size
Dropout	0.1	Regularization in MLP and attention layers
Activation	GELU	Nonlinearity in dense blocks
Optimizer	Adam	With an initial learning rate of 1 × 10^−3^
Batch size	32	Mini-batch size
Epochs	200 (max)	Subject to early stopping
Callbacks	EarlyStopping, ReduceLROnPlateau, ModelCheckpoint	Stability and overfitting prevention

**Table 6:** Model complexity and computational cost.

Model	Params	FLOPs (MFLOPs)	Notes
**CNN–Myo**	99,398	**≈8.1 MFLOPs**	Very lightweight
**CNN–DB3**	109,766	**≈20 MFLOPs**	Higher due to longer inputs
**CViT–Myo**	440,262	**≈10–11 MFLOPs**	Attention over 5 tokens
**CViT–DB3**	441,158	**≈28–30 MFLOPs**	Attention over 19 tokens

**Table 7:** Performance Results of CNN–Myo (Mean ± SD, 95% CI).

Patiente	Test Accuracy	Test Macro-Precision	Test Macro-Recall	Test Macro-F1
P1	0.960 ± 0.000 (±0.000)	0.960 ± 0.000 (±0.000)	0.960 ± 0.000 (±0.000)	0.960 ± 0.000 (±0.000)
P2	0.959 ± 0.0036 (±0.0019)	0.959 ± 0.0036 (±0.0019)	0.959 ± 0.0036 (±0.0019)	0.959 ± 0.0036 (±0.0019)
P3	0.885 ± 0.0052 (±0.0027)	0.885 ± 0.0052 (±0.0027)	0.885 ± 0.0052 (±0.0027)	0.885 ± 0.0052 (±0.0027)
P4	0.980 ± 0.000 (±0.000)	0.980 ± 0.000 (±0.000)	0.980 ± 0.000 (±0.000)	0.980 ± 0.000 (±0.000)
P5	0.960 ± 0.000 (±0.000)	0.960 ± 0.000 (±0.000)	0.960 ± 0.000 (±0.000)	0.960 ± 0.000 (±0.000)
P6	0.913 ± 0.0073 (±0.0038)	0.914 ± 0.0073 (±0.0038)	0.913 ± 0.0073 (±0.0038)	0.913 ± 0.0073 (±0.0038)
P7	0.965 ± 0.0052 (±0.0027)	0.965 ± 0.0052 (±0.0027)	0.965 ± 0.0052 (±0.0027)	0.965 ± 0.0052 (±0.0027)
P8	0.960 ± 0.000 (±0.000)	0.960 ± 0.000 (±0.000)	0.960 ± 0.000 (±0.000)	0.960 ± 0.000 (±0.000)
P9	0.899 ± 0.0036 (±0.0019)	0.899 ± 0.0036 (±0.0019)	0.899 ± 0.0036 (±0.0019)	0.899 ± 0.0036 (±0.0019)
P10	0.950 ± 0.000 (±0.000)	0.950 ± 0.000 (±0.000)	0.950 ± 0.000 (±0.000)	0.950 ± 0.000 (±0.000)

**Table 8:** Performance Results of CNN–NinaPro (Mean ± SD, 95% CI).

Patient	Test Accuracy	Test Macro-Precision	Test Macro-Recall	Test Macro-F1
A1	0.964 ± 0.0048 (±0.0031)	0.964 ± 0.0048 (±0.0031)	0.964 ± 0.0048 (±0.0031)	0.964 ± 0.0048 (±0.0031)
A2	0.858 ± 0.0178 (±0.0119)	0.861 ± 0.0151 (±0.0102)	0.865 ± 0.0163 (±0.0109)	0.858 ± 0.0178 (±0.0119)
A3	0.718 ± 0.0240 (±0.0161)	0.718 ± 0.0189 (±0.0127)	0.717 ± 0.0237 (±0.0159)	0.713 ± 0.0224 (±0.0150)
A4	0.776 ± 0.0417 (±0.0231)	0.788 ± 0.0240 (±0.0133)	0.778 ± 0.0393 (±0.0218)	0.776 ± 0.0442 (±0.0245)
A5	0.834 ± 0.0287 (±0.0159)	0.838 ± 0.0305 (±0.0169)	0.834 ± 0.0287 (±0.0159)	0.828 ± 0.0328 (±0.0181)
A6	0.969 ± 0.0088 (±0.0049)	0.971 ± 0.0064 (±0.0035)	0.969 ± 0.0088 (±0.0049)	0.969 ± 0.0088 (±0.0049)
A7	0.344 ± 0.0112 (±0.0062)	0.344 ± 0.0168 (±0.0093)	0.344 ± 0.0112 (±0.0062)	0.330 ± 0.0146 (±0.0081)
A8	0.816 ± 0.0139 (±0.0077)	0.817 ± 0.0110 (±0.0061)	0.816 ± 0.0139 (±0.0077)	0.813 ± 0.0158 (±0.0087)
A9	0.937 ± 0.0070 (±0.0039)	0.939 ± 0.0046 (±0.0025)	0.936 ± 0.0072 (±0.0040)	0.937 ± 0.0070 (±0.0039)
A10	0.808 ± 0.0201 (±0.0111)	0.826 ± 0.0180 (±0.0100)	0.808 ± 0.0201 (±0.0111)	0.810 ± 0.0189 (±0.0105)
A11	0.936 ± 0.0084 (±0.0048)	0.941 ± 0.0062 (±0.0036)	0.938 ± 0.0089 (±0.0051)	0.939 ± 0.0086 (±0.0050)

**Table 9:** Performance Results of CViT–Myo (Mean ± SD, 95% CI).

Patiente	Test Accuracy	Test Macro-Precision	Test Macro-Recall	Test Macro-F1
P1	0.949 ± 0.0070 (±0.0037)	0.943 ± 0.0152 (±0.0081)	0.901 ± 0.0125 (±0.0067)	0.921 ± 0.0118 (±0.0063)
P2	0.949 ± 0.0034 (±0.0018)	0.901 ± 0.0140 (±0.0075)	0.907 ± 0.0124 (±0.0066)	0.904 ± 0.0094 (±0.0050)
P3	0.858 ± 0.0080 (±0.0042)	0.808 ± 0.0111 (±0.0059)	0.809 ± 0.0173 (±0.0092)	0.809 ± 0.0125 (±0.0067)
P4	0.995 ± 0.0039 (±0.0022)	0.965 ± 0.0125 (±0.0072)	0.982 ± 0.0151 (±0.0087)	0.974 ± 0.0093 (±0.0054)
P5	0.983 ± 0.0039 (±0.0024)	0.960 ± 0.0123 (±0.0074)	0.948 ± 0.0124 (±0.0075)	0.954 ± 0.0101 (±0.0061)
P6	0.977 ± 0.0068 (±0.0036)	0.928 ± 0.0071 (±0.0038)	0.951 ± 0.0122 (±0.0065)	0.939 ± 0.0058 (±0.0031)
P7	0.986 ± 0.0037 (±0.0020)	0.947 ± 0.0115 (±0.0061)	0.948 ± 0.0114 (±0.0061)	0.947 ± 0.0090 (±0.0048)
P8	0.963 ± 0.0064 (±0.0039)	0.959 ± 0.0115 (±0.0069)	0.955 ± 0.0129 (±0.0078)	0.957 ± 0.0110 (±0.0066)
P9	0.969 ± 0.0051 (±0.0027)	0.957 ± 0.0135 (±0.0072)	0.949 ± 0.0135 (±0.0072)	0.953 ± 0.0101 (±0.0054)
P10	0.987 ± 0.0045 (±0.0024)	0.974 ± 0.0139 (±0.0074)	0.940 ± 0.0138 (±0.0074)	0.957 ± 0.0078 (±0.0042)

**Table 10:** Performance Results of CViT–NinaPro (Mean ± SD, 95% CI).

Patiente	Test Accuracy	Test Macro-Precision	Test Macro-Recall	Test Macro-F1
A1	0.977 ± 0.0050 (±0.0029)	0.961 ± 0.0106 (±0.0061)	0.963 ± 0.0120 (±0.0069)	0.962 ± 0.0065 (±0.0037)
A2	0.970 ± 0.0048 (±0.0027)	0.941 ± 0.0141 (±0.0081)	0.954 ± 0.0140 (±0.0081)	0.947 ± 0.0100 (±0.0058)
A3	0.970 ± 0.0043 (±0.0023)	0.960 ± 0.0104 (±0.0055)	0.951 ± 0.0103 (±0.0055)	0.955 ± 0.0070 (±0.0038)
A4	0.975 ± 0.0033 (±0.0018)	0.947 ± 0.0123 (±0.0065)	0.937 ± 0.0105 (±0.0056)	0.942 ± 0.0090 (±0.0048)
A5	0.983 ± 0.0040 (±0.0021)	0.964 ± 0.0102 (±0.0055)	0.961 ± 0.0151 (±0.0080)	0.962 ± 0.0085 (±0.0045)
A6	0.960 ± 0.0043 (±0.0023)	0.931 ± 0.0115 (±0.0061)	0.911 ± 0.0109 (±0.0058)	0.921 ± 0.0063 (±0.0034)
A7	0.663 ± 0.0086 (±0.0046)	0.652 ± 0.0123 (±0.0066)	0.662 ± 0.0126 (±0.0067)	0.657 ± 0.0108 (±0.0057)
A8	0.973 ± 0.0043 (±0.0023)	0.968 ± 0.0146 (±0.0078)	0.930 ± 0.0112 (±0.0060)	0.949 ± 0.0091 (±0.0048)
A9	0.914 ± 0.0093 (±0.0050)	0.885 ± 0.0121 (±0.0064)	0.905 ± 0.0144 (±0.0077)	0.894 ± 0.0102 (±0.0055)
A10	0.971 ± 0.0044 (±0.0024)	0.928 ± 0.0107 (±0.0057)	0.948 ± 0.0090 (±0.0048)	0.938 ± 0.0080 (±0.0043)
A11	0.982 ± 0.0051 (±0.0027)	0.974 ± 0.0119 (±0.0064)	0.935 ± 0.0101 (±0.0054)	0.954 ± 0.0083 (±0.0044)

**Table 11:** Statistical test results for the comparison between CNN and CViT on the Myo and NinaPro datasets.

Comparison	Test	Statistic	*p*-Value	Significant (α = 0.05)
CNN vs. CViT (Myo)	Wilcoxon	W = 8.0	0.049	Sí
CNN vs. CViT (Myo)	t-Student	t = −2.36	0.043	Sí
CNN vs. CViT (NinaPro)	Wilcoxon	W = 21.0	0.320	No
CNN vs. CViT (NinaPro)	t-Student	t = −1.33	0.213	No
Friedman (4 escenarios)	χ^2^ = 4.08	–	0.253	No

**Table 12:** Effect sizes and complementary results of the statistical tests comparing CNN and CViT.

Comparison	Wilcoxon (W, p)	r de Wilcoxon	t-Student (t, p)	Cohen’s d
CNN vs. CViT (Myo)	8.00, *p* = 0.047	−0.63	−2.36, *p* = 0.043	0.75
CNN vs. CViT (NinaPro)	21.00, *p* = 0.286	−0.32	−1.33, *p* = 0.213	0.40

**Table 13:** Statistical comparisons between CNN and CViT, including Bonferroni-adjusted *p*-values.

Comparación	p Original	p Ajustado	Sig. Bonferroni
Wilcoxon (Myo)	0.049	0.196	No
t-Student (Myo)	0.043	0.172	No
Wilcoxon (DB3)	0.320	1.000	No
t-Student (DB3)	0.213	0.852	No

**Table 14:** Comparative results of myoelectric gesture classification methods reported in the literature and in this study.

Method	Accuracy (%)	Dataset/Subjects	Author(s)
CNN (Atzori et al., DB3, 50 gestures)	38.09 ± 14.29%	NinaPro DB3—Amputees, 50 gestures	Gopal et al., 2022 [44]
AtzoriNet RNC	80.46	NinaPro DB3—Amputees (reduced gesture subset)	Atzori et al. [10]
A Multi-Scale CNN (Transfer Learning)	≈97	NinaPro DB2 (healthy)/partial experiments on DB3 (amputees)	Fratti et al., 2024 [19]
Convolutional Neural Network (RNC)	83.7 ± 13.5	NinaPro DB2—Healthy subjects	Asif et al., 2020 [45]
Vision Transformer—TEMGNet	~82.93/82.05	NinaPro DB2—Healthy subjects	Rahimian et al., 2021 [21]
Vision Transformer—EMGTFNet (ViT + FNB)	83.57 ± 3.5	NinaPro DB2—Healthy subjects, 49 gestures	Córdova, Flores & Andreu-Pérez, 2023 [22]
Convolutional Neural Network (RNC)—Myo	94.2 ± 3.2	Proprietary dataset—Amputees, 6 gestures (Myo armband)	This work
Convolutional Neural Network (RNC)—NinaPro DB3	92.0 ± 12.7	NinaPro DB3—Amputees, 6 selected gestures	This work
Vision Transformer—CViT (CNN + ViT)—Myo	96.6 ± 3.9	Proprietary dataset—Amputees, 6 gestures (Myo armband)	This work
Vision Transformer—CViT (CNN + ViT)—NinaPro DB3	94.2 ± 9.3	NinaPro DB3—Amputees, 6 selected gestures	This work

## Data Availability

The datasets generated during the current study (Myo–Amputee cohort and processed files) have been deposited in Mendeley Data and are publicly available at [DOI: 10.17632/tgtdthtxf5.1]. The NinaPro DB3 dataset analyzed in this work is publicly available from the provider’s repository. All data supporting the findings of this study are available at the links above.

## Abbreviations

The following abbreviations are used in this manuscript:

sEMG	Surface Electromyography
EMG	Electromyography
HD-sEMG	High-Density Surface Electromyography
IMU	Inertial Measurement Unit
Myo/MYB	Myo armband
CSV	Comma-Separated Values
NinaPro (DB1/DB2/DB3)	Non-Invasive Adaptive Hand Prosthetics Database
BioPatRec	Biopattern Recognition for Prosthetic Control
CapgMyo	Capital Normal University Gesture sEMG Database
SVM	Support Vector Machine
LDA	Linear Discriminant Analysis
CSL-HD	Control and Simulation Laboratory—High Density sEMG Dataset
CNN	Convolutional Neural Network
ViT	Vision Transformer
CViT	Convolutional Vision Transformer
RNN	Recurrent Neural Network
LSTM	Long Short-Term Memory
BiLSTM	Bidirectional Long Short-Term Memory
3D-CNN	Convolutional Neural Networks (CNNs)
MLP	Multilayer perceptron
U-Net	Convolutional Network for Biomedical Image Segmentation
MobileNetV2	Mobile Neural Network Version 2
EMGNet	Electromyography Neural Network
TEMGNet	Temporal Electromyography Neural Network
EMGTFNet	Electromyography Temporal-Frequency Network
BSS-CViT	Broad-Spectrum Spatial Convolutional Vision Transformer
TraHGR	Transformer-based Hand Gesture Recognition
ViT-HGR	Vision Transformer for Hand Gesture Recognition
CT-HGR	Convolutional Transformer for Hand Gesture Recognition
MHSA	Multi-head self-attention
LayerNorm	Layer Normalization
GELU	Gaussian Error Linear Unit
ReLU	Rectified Linear Unit
Softmax	Softmax Function
Dropout	regularization method that randomly deactivates neurons during training to reduce overfitting
Adam	Adaptive Moment Estimation
SGD	Stochastic Gradient Descent
F1-score	harmonic mean of precision and recall, also called the F-measure at β = 1
SD	Standard Deviation
CI	Confidence Interval
95% CI	95% Confidence Interval
t	Student’s *t*-test
train–val	training–validation split
k-fold	k-fold cross-validation
DASH	Disabilities of the Arm, Shoulder and Hand
RE	Rest
AP	Power Grip
PI	Palm In
PO	Palm Out
MO	Open Hand
AT	Pinch Grip
BCI	Brain–Computer Interface

## Appendix A

**Table A1:** Results of statistical tests comparing CNN and CViT on the Myo and NinaPro datasets, excluding Patient 7.

Comparison	Test	Statistic	*p*-Value	Significant (α = 0.05)
CNN vs. CViT (Myo)	Wilcoxon	W = 8.0	0.049	Yes
CNN vs. CViT (NinaPro, without P7)	Wilcoxon	W = 21.0	0.557	No
Friedman (4 scenarios, without P7)	χ^2^	3.80	0.284	No

## References

[1] Rezaee K., Khavari S.F., Ansari M., Zare F., Roknabadi M.H.A. (2024). Hand Gestures Classification of sEMG Signals Based on BiLSTM-Metaheuristic Optimization and Hybrid U-Net–MobileNetV2 Encoder Architecture. Sci. Rep..

[2] Jiang B., Wu H., Xia Q., Xiao H., Peng B., Wang L., Zhao Y. (2024). An Efficient Surface Electromyography-Based Gesture Recognition Algorithm Based on Multiscale Fusion Convolution and Channel Attention. Sci. Rep..

[3] Emimal M., Hans W.J., Inbamalar T.M., Lindsay N.M. (2025). Classification of EMG Signals with CNN Features and Voting Ensemble Classifier. Comput. Methods Biomech. Biomed. Eng..

[4] Zhang X., Qu Y., Zhang G., Wang Z., Chen C., Xu X. (2025). Review of sEMG for Exoskeleton Robots: Motion Intention Recognition Techniques and Applications. Sensors.

[5] Lee Y.J., Park C., Kim H., Cho S.J., Yeo W.-H. (2024). Artificial Intelligence on Biomedical Signals: Technologies, Applications, and Future Directions. Med-X.

[6] Jeong I., Chung W.G., Kim E., Park W., Song H., Lee J., Oh M., Kim E., Paek J., Lee T. (2025). Machine Learning in Biosignal Analysis from Wearable Devices. Mater. Horiz..

[7] Anwar A., Khalifa Y., Coyle J.L., Sejdic E. (2025). Transformers in Biosignal Analysis: A Review. Inf. Fusion.

[8] Gopi V. (2025). Lightweight Transformer Models for Biomedical Signal Processing: Trends, Challenges, and Future Directions. Sciety. https://sciety-labs.elifesciences.org/articles/by?article_doi=10.21203/rs.3.rs-7620509/v1.

[9] Farmani J., Bargshady G., Gkikas S., Tsiknakis M., Fernández Rojas R. (2025). A CrossMod-Transformer Deep Learning Framework for Multi-Modal Pain Detection through EDA and ECG Fusion. Sci. Rep..

[10] Atzori M., Cognolato M., Müller H. (2016). Deep Learning with Convolutional Neural Networks Applied to Electromyography Data: A Resource for the Classification of Movements for Prosthetic Hands. Front. Neurorobot..

[11] Chen L., Fu J., Wu Y., Li H., Zheng B. (2020). Hand Gesture Recognition Using Compact CNN via Surface Electromyography Signals. Sensors.

[12] Hu Y., Wong Y., Wei W., Du Y., Kankanhalli M., Geng W. (2018). A Novel Attention-Based Hybrid CNN–RNN Architecture for sEMG-Based Gesture Recognition. PLoS ONE.

[13] Zhou Z., Tao Q., Su N., Liu J., Chen Q., Li B. (2024). Lower Limb Motion Recognition Based on sEMG and CNN–TL Fusion Model. Sensors.

[14] Bai A., Song H., Wu Y., Dong S., Feng G., Jin H. (2025). Sliding-Window CNN + Channel-Time Attention Transformer Network Trained with Inertial Measurement Units and Surface Electromyography Data for the Prediction of Muscle Activation and Motion Dynamics. Sensors.

[15] Montazerin M., Rahimian E., Naderkhani F., Atashzar S.F., Yanushkevich S., Mohammadi A. (2023). Transformer-Based Hand Gesture Recognition from High-Density EMG Signals. Sci. Rep..

[16] Shen S., Wang X., Mao F., Sun L., Gu M. (2022). Movements Classification through sEMG with Convolutional Vision Transformer and Stacking Ensemble Learning. IEEE Sens. J..

[17] Xia Y., Qiu D., Zhang C., Liu J. (2025). sEMG-Based Gesture Recognition Using Multi-Stream Adaptive CNNs with Integrated Residual Modules. Front. Bioeng. Biotechnol..

[18] Shin J., Miah S.M., Konnai S., Takahashi I., Hirooka K. (2024). Hand Gesture Recognition Using sEMG Signals with a Multi-Stream Time-Varying Feature Enhancement Approach. Sci. Rep..

[19] Fratti R., Marini N., Atzori M., Müller H., Tiengo C., Bassetto F. (2024). A Multi-Scale CNN for Transfer Learning in sEMG-Based Hand Gesture Recognition for Prosthetic Devices. Sensors.

[20] Khan A., Rauf Z., Khan A.R., Rathore S. (2025). A Recent Survey of Vision Transformers for Medical Image Segmentation. IEEE Access.

[21] Rahimian E., Zabihi S., Asif A., Farina D., Atashzar S.F., Mohammadi A. (2021). TEMGNet: Deep Transformer-Based Decoding of Upper-Limb sEMG for Hand Gesture Recognition. arXiv.

[22] Córdova J.C., Flores C., Andreu-Perez J. (2023). EMGTFNet: Fuzzy Vision Transformer to Decode Upper-Limb sEMG Signals for Hand Gesture Recognition. arXiv.

[23] Dere M.D., Lee B. (2023). A Novel Approach to Surface EMG-Based Gesture Classification Using a Vision Transformer Integrated with Convolutive Blind Source Separation. IEEE J. Biomed. Health Inform..

[24] Zabihi S., Rahimian E., Asif A., Mohammadi A. (2022). TraHGR: Transformer for Hand Gesture Recognition via Electromyography. arXiv.

[25] Atzori M., Gijsberts A., Castellini C., Caputo B., Mittaz Hager A.G., Elsig S., Giatsidis G., Bassetto F., Müller H. (2014). Electromyography Data for Non-Invasive Naturally Controlled Robotic Hand Prostheses. Sci. Data.

[26] Ninapro. https://ninapro.hevs.ch/instructions/DB3.html.

[27] Benalcázar M.E., Motoche C., Zea J.A., Jaramillo A.G., Anchundia C.E., Zambrano P. Real-Time Hand Gesture Recognition Using the Myo Armband and Muscle Activity Detection. Proceedings of the IEEE ETCM.

[28] Xiong D., Zhang D., Zhao X., Zhao Y. (2021). Deep Learning for EMG-Based Human–Machine Interaction: A Review. IEEE/CAA J. Autom. Sin..

[29] Phinyomark A., Scheme E. (2018). EMG Pattern Recognition in the Era of Big Data and Deep Learning. Big Data Cogn. Comput..

[30] Lee K.H., Min J.Y., Byun S. (2022). Electromyogram-Based Classification of Hand and Finger Gestures Using Artificial Neural Networks. Sensors.

[31] Karnam K., Dubey S.R., Turlapaty A.C., Gokaraju B. (2022). EMGHandNet: A Hybrid CNN and Bi-LSTM Architecture for Hand Activity Classification Using Surface EMG Signals. Biocybern. Biomed. Eng..

[32] Tovar M.T., Rivera Gómez A.E., Rodríguez Serrezuela R. Feature-DT-DF-EMG-UC Software: Best Patient Selection on biomedical signals with multimodal time and frequency analysis. Proceedings of the IEEE AmITIC.

[33] Chen C., Chen Z., Zhou Y., Hao Y., Peng B., Xie X., Xie H. (2024). A Reliable Evaluation Approach for Multichannel Signal Denoising Algorithms Based on a Novel Arterial Pulse Acquisition System. Heliyon.

[34] Montazerin M., Zabihi S., Rahimian E., Mohammadi A., Naderkhani F. (2022). ViT-HGR: Vision Transformer-Based Hand Gesture Recognition from high density surface EMG signals. arXiv.

[35] Li Q., Cao W., Zhang A. (2025). Multi-Stream Feature Fusion of Vision Transformer and CNN for Precise Epileptic Seizure Detection from EEG Signals. J. Transl. Med..

[36] Godoy R.V., Dwivedi A., Liarokapis M. (2022). Electromyography-Based Decoding of Dexterous In-Hand Manipulation Motions with Temporal Multichannel Vision Transformers. IEEE Trans. Neural Syst. Rehabil. Eng..

[37] Le D.P.C., Wang D., Le V.-T. (2024). A Comprehensive Survey of Recent Transformers in Image, Video and Diffusion Models. Comput. Mater. Contin..

[38] Moslhi A.M., Aly H.H., ElMessiery M. (2024). The Impact of Feature Extraction on Classification Accuracy Using a Signal Transformer for EMG. Sensors.

[39] Hadjiiski L., Cha K., Chan H.-P., Drukker K., Morra L., Näppi J.J., Sahiner B., Yoshida H., Chen Q., Deserno T.M. (2023). AAPM Task Group Report 273: Recommendations on best practices for AI and machine learning for computer-aided diagnosis in medical imaging. Med. Phys..

[40] Chae A., Yao M.S., Sagreiya H., Goldberg A.D., Chatterjee N., MacLean M.T., Duda J., Elahi A., Borthakur A., Ritchie M.D. (2024). Strategies for Implementing Machine Learning Algorithms in Clinical Radiology. Radiology.

[41] Wang J., Liu N., Xie Y., Que S., Xia M. (2025). A Multimodal CNN–Transformer Network for Gait Pattern Recognition with Wearable Sensors in Weak GNSS Scenarios. Electronics.

[42] Yuan Y., Liu J., Dai C., Liu X., Hu B., Fan J. (2024). Exploring Pattern-Specific Components Associated with Hand Gestures Through Different sEMG Measures. J. NeuroEng. Rehabil..

[43] Aghchehli E., Jabbari M., Ma C., Dyson M., Nazarpour K. (2025). Medium Density EMG Armband for Gesture Recognition. Front. Neurorobot..

[44] Gopal P., Gesta A., Mohebbi A. (2022). A Systematic Study on Electromyography-Based Hand Gesture Recognition for assistive robots using deep learning and machine learning models. Sensors.

[45] Asif A.R., Waris A., Gilani S.O., Jamil M., Ashraf H., Shafique M., Niazi I.K. (2020). Performance Evaluation of convolutional neural network for hand gesture recognition Using EMG. Sensors.

[46] Tam S., Boukadoum M., Campeau-Lecours A., Gosselin B. (2021). Intuitive Real-Time Control Strategy for high-density myoelectric hand prosthesis using deep and transfer learning. Sci. Rep..

[47] Wang H., Li N., Gao X., Jiang N., He J. (2024). Analysis of Electrode Locations on Limb Condition Effect for Myoelectric Pattern Recognition. J. NeuroEng. Rehabil..

[48] Pan L., Zhang D., Jiang N., Sheng X., Zhu X. (2015). Improving Robustness Against Electrode Shift of HD-EMG for Myoelectric Control through common spatial patterns. J. NeuroEng. Rehabil..

